# The Interplay Between Non-coding RNAs and Insulin-Like Growth Factor Signaling in the Pathogenesis of Neoplasia

**DOI:** 10.3389/fcell.2021.634512

**Published:** 2021-03-09

**Authors:** Soudeh Ghafouri-Fard, Atefe Abak, Mahdi Mohaqiq, Hamed Shoorei, Mohammad Taheri

**Affiliations:** ^1^Department of Medical Genetics, Shahid Beheshti University of Medical Sciences, Tehran, Iran; ^2^Department of Medical Genetics, Faculty of Medicine, Tabriz University of Medical Sciences, Tabriz, Iran; ^3^School of Advancement, Centennial College, Ashtonbee Campus, Toronto, ON, Canada; ^4^Wake Forest Institute for Regenerative Medicine, School of Medicine, Wake Forest University, Winston-Salem, NC, United States; ^5^Department of Anatomical Sciences, Faculty of Medicine, Biranjd University of Medical Sciences, Birjand, Iran; ^6^Urology and Nephrology Research Center, Shahid Beheshti University of Medical Sciences, Tehran, Iran

**Keywords:** IGF, miRNA, lncRNA, expression, disorders

## Abstract

The insulin-like growth factors (IGFs) are polypeptides with similar sequences with insulin. These factors regulate cell growth, development, maturation, and aging via different processes including the interplay with MAPK, Akt, and PI3K. IGF signaling participates in the pathogenesis of neoplasia, insulin resistance, diabetes mellitus, polycystic ovarian syndrome, cerebral ischemic injury, fatty liver disease, and several other conditions. Recent investigations have demonstrated the interplay between non-coding RNAs and IGF signaling. This interplay has fundamental roles in the development of the mentioned disorders. We designed the current study to search the available data about the role of IGF-associated non-coding RNAs in the evolution of neoplasia and other conditions. As novel therapeutic strategies have been designed for modification of IGF signaling, identification of the impact of non-coding RNAs in this pathway is necessary for the prediction of response to these modalities.

## Background

**FIGURE 1:**
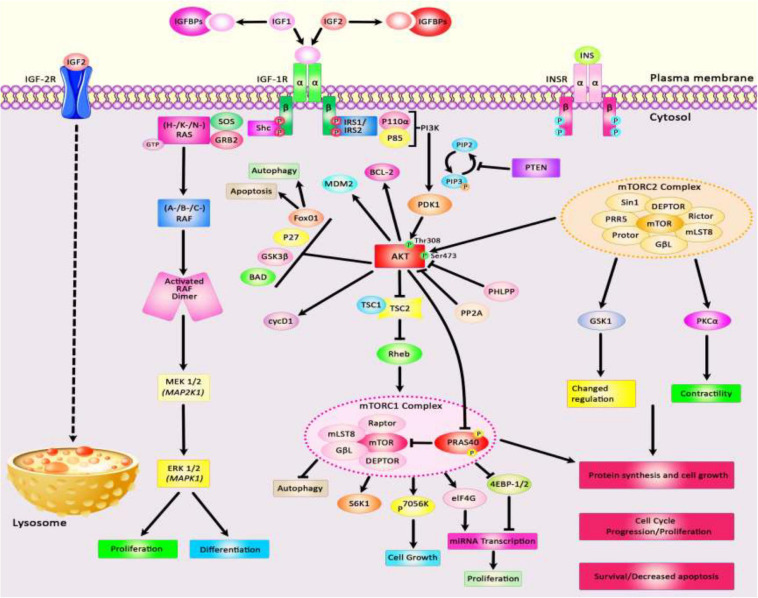
A schematic diagram of the insulin-like growth factor (IGF) signal transduction and main downstream effects. Both IGF-1 and IGF-2 could bind with plasma membrane IGF-1R and IGF-1/Insulin hybrid receptor leading to autophosphorylation of this target receptor in the intracellular β-subunits and thus triggering the catalytic function of the IGF-1R, while insulin binds only to the Insulin-R. IGFBPs could regulate the bioavailability of both IGF-1 and IGF-2 signaling cascades. The bioavailability of IGF-2 could also be regulated via binding to the IGF-2R which results in receptor-triggered internalization and endosomal degradation of IGF-2 in the lysosomes. Phosphorylated β-subunits could in turn create docking sites for the adaptor proteins IRS-1/2, and Shc that modulate the activation of two signaling pathways: PI3K/AKT and MAPK/ERK. Activation of the PI3K and AKT pathway leads to modulation of a variety of cell signaling cascades, such as regulation of TSC1/2 to suppress mTORC1 complex and modulation of 4EB-P1 and S6K1/2 phosphorylation, promoting cell survival via activation or suppression of major effectors like the Foxo transcription factors, BCL-2, BAD, and P27, upregulation of transformation of glucose to glycogen through suppression of GSK-3β, increasing protein synthesis, as well as suppressing apoptosis and autophagy. Activation of AKT family of kinases via PDK1 and mTORC2 leads to the phosphorylation at Thr308 and Ser473, respectively. Besides, docking of Grb2 to the phosphorylated IGF-1R β subunits could trigger the Ras/Raf/MEK/ERK axis. Shc binding to activated IGF-1R leads to stimulation of the MAPK/ERK cascade which regulates a kinase signaling pathway and eventually results in promoting cellular proliferation via enhancing transcription factors activities including ELK1.

The insulin-like growth factors (IGFs) are involved in growth and developmental processes and are evolutionarily conserved among several species (Rosenzweig, 2020). The functions of IGFs are mediated through two receptor tyrosine kinases and receptors for IGF1 and insulin. Besides, several IGF binding proteins selectively inhibit IGF1 or IGF2. IGF1 receptors have been shown to be up-regulated in tumors, thus participating in the tumorigenesis, resistance to therapies, and facilitation of metastasis in various cancer kinds (Rosenzweig, 2020). IGF1 receptors are known inducers of the Akt and mitogen-activated protein kinase (MAPK) (Pollak, 2008). Besides, IGF signaling is involved in the pathogenesis of insulin resistance and other disorders (Rosenzweig, 2020). The contribution of IGF in the pathogenesis of a wide assortment of human disorders including neoplasia and other disorders is explained by its influence on energy metabolism and cell growth (Pollak, 2008). IGF1 acts downstream of the growth hormone and through activation of MAPK and PI3K pathways and anabolism, it promotes growth and maturation of almost all tissues. Therefore, it is also involved in the aging process (Wrigley et al., 2017). Figure 1 depicts an overview of Insulin-like growth factor (IGF) signal transduction and two downstream signaling pathways: PI3K/AKT and MAPK/ERK. The IGF signaling network is composed of three receptor tyrosine kinases (IGF1R, IGF2R, and INSR), three ligands (insulin, IGF1, and IGF2), and six serum insulin-like growth factor binding proteins (IGFBP). Figure 1 shows the IGF signal transduction and its downstream effectors.

Recent investigations have verified the influence of regulatory non-coding RNAs on IGF signaling (Chen B. et al., 2019). Most investigations in this regard have focused on long non-coding RNAs (lncRNAs) and microRNAs (miRNAs) (Chen B. et al., 2019). LncRNAs are transcripts with sizes of more than 200 nucleotides which are principally produced by RNA polymerase II. These transcripts have various functions in the modulation of genomic structure, chromatin configuration, mRNA stability, alternative splicing, and enhancement or inhibition of transcription. The other types of regulatory non-coding RNAs, i.e., miRNAs mainly influence gene expression at the post-transcriptional phase via binding with the 3′ UTR of their specific targets. Both classes of non-coding RNAs participate in the pathogenesis of human diseases. We designed the current study to search the available data about the role of IGF-associated non-coding RNAs in the evolution of neoplasia and other conditions.

## IGF-Associated miRNAs in Human Disorders

Several IGF-associated miRNAs have been dysregulated in neoplastic conditions. For instance, experiments in ovarian cancer cells have shown that miR−19a−3p suppresses the levels of IGF binding protein−3 (IGFBP−3), thus promoting the growth and migration of these cells. Notably, the expression of this miRNA can be modulated by NF−κB (Bai et al., 2019). Shastri et al. have demonstrated the inhibitory effects of the miR-29 family on IGF-1. Members of the miR-30 family can inhibit both IGF-1 and IGF-1R. Notably, calorie restriction has resulted in the over-expression of miR-29 and miR-30 in the normal liver and the liver being metastasized by breast cancer cells, indicating a possible role for dietary modifications in the management of liver metastases (Shastri et al., 2020). In nasopharyngeal squamous cell carcinoma cells, miR-30a inhibitor could reverse IGF-I-associated epithelial-mesenchymal transition (EMT). The IGF-1R/Src/miR-30a/E-cadherin axis has been identified as an important pathway in the regulation of EMT in these cells (Wang et al., 2016). miR-99a is another miRNA that can inhibit proliferation, migration, and invasion of breast carcinoma via suppression of IGF-1R (Xia et al., 2016). Being up-regulated in hepatocellular carcinoma cells, miR-155 can increase expression of IGF-II and IGF-1R, while decreasing IGFBP-3 expression. Through these pathways, miR-155 can increase proliferation, migration, and clonogenicity of hepatocellular carcinoma cells (El Tayebi et al., 2015). In the same type of cancer, miR-342-3p can inhibit cell proliferation through the suppression of the IGF-1R-associated Warburg effect (Liu et al., 2018d). In colorectal cancer cells, the oncogenic protein IGF2BP2 has a functional interaction with miR-195 through which it regulates RAF1 expression and participates in the carcinogenic process (Ye S. et al., 2016). Meanwhile, miR-197 can inhibit the expression of IGFBP3 through binding with its 3′-UTR, hence enhancing cell migratory potential and invasion of colorectal cancer cells (Zhou et al., 2018). Supplementary Table 1 reviews the results of studies that displayed the role of IGF-associated miRNAs in the neoplastic conditions.

**FIGURE 2:**
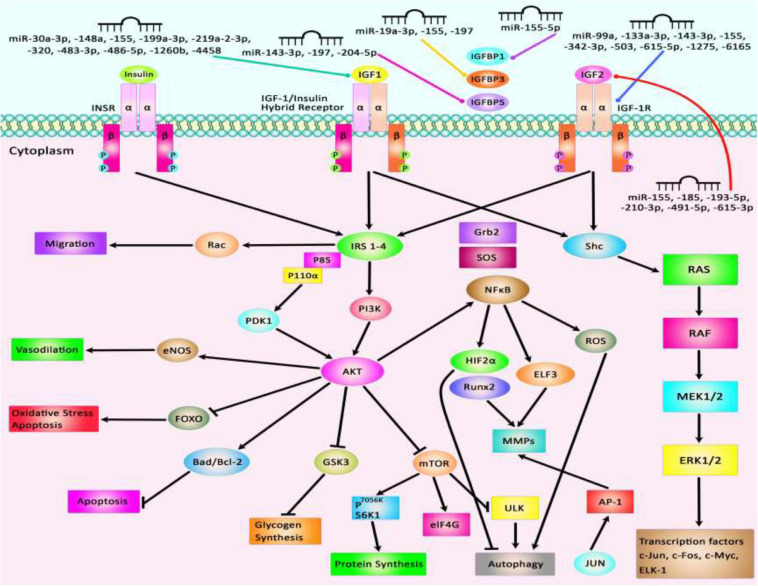
Several proteins in the IGF signaling pathway such as IGF1, IGF2, IGF-1R, and IGFBPs are regulated by miRNAs. Through modulation of these proteins, miRNAs can affect several cellular processes such as apoptosis, autophagy, protein synthesis, response to oxidative stress, and cell migration.

The interaction between IGF-related proteins and miRNAs has been also assessed in non-neoplastic conditions. For instance, IGF-1 is targeted by miR-17. This miRNA has been over-expressed in ox-LDL treated human umbilical vascular endothelial cells (HUVECs) in association with down-regulation of IGF-1. Up-regulation of miR-17 has enhanced cell viability and suppressed the apoptosis of ox-LDL exposed cells. Such effects have been accompanied by down-regulation of Bax and Caspase3 expressions, while up-regulation of Bcl-2, suggesting a role for miR-17 as a biomarker for coronary heart disease (Chen Z. et al., 2019). Expression of miR-30a-3p has been elevated in the placenta samples of women with preeclampsia. This miRNA has been shown to regulate the expression of IGF-1, therefore influencing the invasive capacity and apoptosis of trophoblasts (Niu et al., 2018). Over-expression of miR-129 has suppressed proliferation and migration of Schwan cells and axonal outgrowth of dorsal root ganglion neurons through modulation of several targets including IGF-1 (Zhu H. et al., 2018). Yang et al. have reported up-regulation of miR-143-3p in synovial tissues of patients with rheumatoid arthritis compared with those affected with osteoarthritis. Down-regulation of miR-143-3p has inhibited cell proliferation, enhanced apoptosis, and reduced production of inflammatory cytokines. miR-143-3p has been shown to target IGF1R and IGFBP5 and regulate the Ras/p38 MAPK axis (Liu et al., 2018c). In colonic smooth muscle cells, miR-155 has been shown to down-regulate IGF-1 levels. Up-regulation of miR-155 has increased apoptosis of these cells and reduced the thickness of the related tissue in the diabetic mice, suggesting the role of this miRNA in the aggravation of colonic dysmotility (Shen et al., 2020). Figure 2 illustrates the IGF signaling cascade modulating by dysregulated miRNAs in various human diseases as well as cancers. Table 1 reviews the role of IGF-associated miRNAs in non-neoplastic conditions.

**TABLE 1:** IGF-associated miRNAs in non-malignant disorders.

Type of disease	microRNA	*P*-value	Animal	Clinical samples (human)	Cell lines	Target	Pathway	Function	References
Coronary Heart Disease (CHD)	miR-17	<0.01	–	–	HUVECs	IGF-1, Caspase-3, Bax, Bcl-2	–	Overexpression of miR-17 via targeting IGF-1 could promote the proliferation and inhibit apoptosis of HUVECs.	Chen Z. et al., 2019
Diabetic Retinopathy (DR)	miR-18b	<0.01	–	–	HRECs	IGF-1	AKT, MEK, ERK	Downregulation of miR-18b by targeting IGF-1 could increase the proliferation of HRECs exposed to VEGF secretion and normal glucose.	Wu et al., 2016
Polycystic Ovary	miR-19b	<0.01	–	PCOS (*n* = 18), normally menstruating women (*n* = 10)	KGN	IGF-1, CDK1, Cyclin-D1	–	Downregulation of miR-19b by targeting IGF-1 could enhance ovarian GCs proliferation in PCOS.	Zhong et al., 2018
Preeclampsia (PE)	miR-30a-3p	<0.05	–	PE (*n* = 25), normal pregnant women (*n* = 20)	HTR-8/SVneo, JEG-3	IGF-1	–	Overexpression of miR-30a-3p via targeting IGF-1 could induce the apoptosis of trophoblast HTR-8/SVneo cells.	Niu et al., 2018
Polycystic Ovary Syndrome (PCOS)	miR-99a	<0.05	–	15 pairs of married women with PCOS and a control group of women without PCOS	COV434	IGF-1R	–	Overexpression of miR-99a by targeting IGF-1R could reduce the proliferation and promote apoptosis of human granulosa cells (GCs).	Geng et al., 2019
Peripheral Nerve Injury (PNI)	miR-129	<0.01	Male SD rats	–	SCs, 293T	IGF-1	–	Overexpression of miR-129 by targeting IGF-1 could suppress the proliferation and migration of SCs, and axonal outgrowth of DRG neurons in PNI.	Zhu H. et al., 2018
Rheumatoid Arthritis (RA)	miR-129-5p	<0.05	–	RA (*n* = 15), healthy controls (*n* = 12)	FLSs	IGF-1R, Caspase-3/8	Src/ERK/Egr-1	Overexpression of miR-129-5p by targeting IGF-1R and activating Src/ERK/Egr-1 signaling could inhibit cell proliferation and induce apoptosis of RA cells.	Zhang Y. et al., 2019
Idiopathic Pulmonary Fibrosis (IPF)	miR-130b-3p	<0.05	–	4 IPF patients and 3 normal lung tissues	A549, ATII, MRC5	IGF-1	–	Downregulation of miR-130b-3p by enhancing IGF-1 production from the epithelium of the lung could activate fibroblasts to increase the proliferation, migration ability, and expression of collagen I of fibroblasts in co-culture systems.	Li S. et al., 2016
Diabetic Retinopathy (DR)	miR-142-5p	<0.001	Male SD rats	–	HRECs, 293T	IGF-1, VEGF	PI3K, ERK, AKT, VEGF	Inhibition of miR-142-5p via blocking the IGF-1/p-IGF-1R pathway could promote HREC proliferation in response to DR conditions.	Qiao et al., 2020
Rheumatoid Arthritis (RA)	miR-143-3p	<0.01	–	5 pairs of RA and normal control	MH7A	IGF-1R, IGFBP-5, TNF-α, Bax, Bcl-2, Caspase-3	Ras/p38 MAPK	Downregulation of miR-142-3p could reduce cell proliferation and promotes cell apoptosis by targeting IGF-1R and IGFBP-5.	Liu et al., 2018c
Fracture Healing	miR-148a	<0.0 1	Male Wistar rats	–	293T, rBMSCs	IGF-1, Runx2, OCN, OPN	–	Downregulation of miR−148a by targeting IGF-1 could promote the expression of osteogenesis−related proteins and regulate bone BMSCs−mediated fracture healing.	Liu et al., 2018a
Diabetes Mellitus	miR-155	<0.05	–	–	SMCs	IGF-1	–	Overexpression of miR-155 by regulating the IGF-1 could decrease the thickness of colonic smooth muscle tissues in diabetic mice and also could increase apoptosis of colonic SMCs.	Shen et al., 2020
Non-alcoholic Fatty Liver Disease (NAFLD)	miR-190b	<0.05	Male C57Bl/6 mice	15 pairs of NAFLD and NNTs	L02	IGF-1, ADAMTS9	IRS2/AKT	Downregulation of miR-190b by directly targeting IGF-1 and ADAMTS9 could regulate lipid metabolism and insulin signaling pathway *in vitro* and could reduce the hepatic steatosis and insulin resistance *in vivo*.	Xu et al., 2018
Diabetic Cardiomyopathy	miR-193-5p	<0.05	Wistar rat	–	MMEC	IGF-2	–	Downregulation of miR-193a-5p by inhibiting IGF-2 could reduce cell migration and proliferation in type 2 diabetic cardiomyopathy.	Yi et al., 2017
Estrogen-Mediated Autophagy	miR-199a-3p	<0.05	–	–	MLO-Y4, MC3T3-E1	IGF-1	mTOR	Overexpression of miR-199a-3p by targeting IGF-1 and inhibiting the mTOR signaling pathway could induce autophagy in osteocyte-like MLO-Y4 cells.	Fu et al., 2018
Atherosclerosis	miR-210-3p	<0.01	Male C57BL/6J mice	–	THP-1	IGF-2, TNF-α, MCP-1, IL-6, iNOS	NF-kB	Overexpression of miR-210-3p by inhibiting the IGF-2/IGF-2R axis could inhibit the expression of CD36 and NF-κB, then led to a reduction in the inflammatory response of macrophages and lipid accumulation in atherosclerosis.	Qiao et al., 2020
Spinal Cord Injury (SCI)	miR-219a-2-3p	<0.01	Female SD rats	–	NSCs, PC12	IFG-1, BAX, Bcl-2, Beclin-1, Caspase-3	NF-kB	Exposure of exosomes derived from NSCs to IGF-1 via the miR-219a-2-3p-dependent pathway could suppress the nerve inflammation, inhibit apoptosis, and promote nerve regeneration after the SCI.	Moharamoghli et al., 2019
Acute Myocardial Infarction (AMI)	miR-320	<0.05	Female Wistar rats	–	–	IGF-1, ASK1, Bcl-2, Bax, Caspase-3	p38, JNK	Downregulation of miR-320 via increasing IGF-1 could suppress cardiomyocyte apoptosis.	Song et al., 2016
Cerebral I/R Injury	miR-320	<0.01	Male C57BL mice	–	PC12	IGF-1	–	overexpression of miR-320 by targeting IGF-1 could enhance brain infarction volume and edema volume in MCAO/R mice.	Liang et al., 2018
Polycystic Ovary Syndrome (PCOS)	miR-323-3p	<0.05	–	20 pairs of PCOS lesion and normal ovarian cortex tissue samples	KGN, CCs	IGF-1, AR, AMHR-II, CYP19A, EGFR, GATA-4	–	Downregulation of miR-323-3p by targeting IGF-1 could enhance apoptosis and increase the steroidogenesis in KGN cells.	Wang et al., 2019b
PCOS	miR-483	<0.001	–	20 pairs of PCOS lesion and normal ovarian cortex tissue samples	KGN	IGF-1, CCNB1, CCND1, CDK2	–	Overexpression of miR-483 possibly by targeting IGF-1 could inhibit KGN cell viability and proliferation and induces cell cycle arrest.	Xiang et al., 2016
Rheumatoid Arthritis (RA)	miR-483-3p	<0.001	–	Synovial tissues from RA patients (*n* = 10), healthy controls (*n* = 6)	HFLS, HFLS-RA	IGF-1	–	Overexpression of miR-483-3p via targeting IGF-1 could promote cell proliferation, the G0/G1-to-S phase transition, and suppress apoptosis in RA FLSs.	Wang Y. et al., 2020
Acute Myocardial Infarction (AMI)	miR-483-3p	<0.05	–	6 pairs of AMI patients and normal volunteers	H9c2	IGF-1, Bax, Bcl-2, Caspase-3, Caspase-9	–	Overexpression of miR-483-3p by targeting IGF-1 could promote apoptosis in the AMI model.	Sun et al., 2018
Congenital Heart Disease	miR-486-5p	<0.01	–	–	H9C2	IGF-1, Bcl-2, Bax, Caspase-3, Caspase-9,	–	Downregulation of miR-486-5p by targeting IGF-1 could increase cardiomyocyte growth in hypoxic conditions.	Fan et al., 2019
Coronary Heart Disease (CHD)	miR-503	<0.05	–	–	H9c2	IGF-1R, Cyto-C, c-PARP, Caspase3	PI3K/AKT	Overexpression of miR-503 by inhibiting the PI3K/AKT pathway via targeting IGF-1R could accelerate hypoxia-induced injury.	Zhu W. et al., 2018
Lumbar Disc Degeneration (LDD)	miR-4458	<0.05	–	24 LDD samples and 22 normal controls	SV40	IGF-1	PI3K/AKT	Overexpression of miR-4458 by decreasing both total IGF-1R and phosphorylated IGF-1R could lead to a decrease of phosphorylated AKT. Also, miR-4458 by suppressing the PI3K/AKT pathway via inhibiting IGF-1R could promote the development of LDD.	Liu Z. Q. et al., 2016

Overexpression of IGF-associated miRNAs namely miR-30a-3p, miR-155, miR-199a-3p, and miR-486-5p has important roles in different conditions such as preeclampsia, hepatocellular carcinoma, estrogen-mediated autophagy, and congenital heart disease (El Tayebi et al., 2015; Fu et al., 2018; Niu et al., 2018; Fan et al., 2019). Besides, dysregulation of miR-210-3p, miR-491-5p, and miR-615-3p contributes to the pathogenesis of atherosclerosis, colorectal carcinoma, and non-small lung cancer through modulation of IGF2 expression level (Liu j. et al., 2019; Lu et al., 2019; Qiao et al., 2020). Besides, aberrant expressions of miR-204-5p, miR-197, and miR-155-5p participate in the pathogenesis of papillary thyroid carcinoma, colorectal cancer, and non-small lung cancer through affecting expressions of IGFBP5, IGFBP3, and IGFBP1, respectively (Ling et al., 2015; Liu L. et al., 2015; Zheng et al., 2018). miR-99a, miR-503, and miR-1275 contribute to the pathogenesis of polycystic ovary syndrome, coronary heart disease, and hepatocellular carcinoma by affecting IGF-1R levels (Fawzy et al., 2015; Zhu W. et al., 2018; Geng et al., 2019). Figure 2 summarizes the role of a number of IGF-associated miRNAs in human disorders including cancers.

## IGF-Associated lncRNAs in Human Disorders

Several lncRNAs have functional links with IGF-related proteins. Wang et al. have demonstrated over-expression of circ_0014130 in Non-Small Cell Lung Carcinoma tissues and cells. Down-regulation of this cirCRNA has suppressed cell proliferation and enhanced cell apoptosis in these cells. Circ_0014130 has functional interactions with miR-142-5p and IGF-1. Small interfering RNA-mediated circ_0014130 silencing has enhanced IGF-1 levels through up-regulation of miR-142-5p (Wang M. et al., 2020). Another study in this kind of cancer has shown up-regulation of HOXA-AS2 in the tumor samples. HOXA-AS2 silencing has decreased the expression of IGF2. Therefore, HOXA-AS2 promotes the migratory and invasive capacities of lung cancer cells by enhancing IGF2 expression (Zheng et al., 2019). In cervical cancer cells, the expression of linc00319 has been increased. Linc00319 silencing has suppressed cell proliferation, invasion, and migration of cervical cancer cells. This lncRNA interacts with miR-147a to modulate the expression of IGF1R (Ma et al., 2020). DBH-AS1 is another oncogenic lncRNA in hepatocellular carcinoma. Up-regulation of this lncRNA has been associated with the down-regulation of miR-138. DBH-AS1 knockdown and miR-138 up-regulation have decreased cell viability, repressed colony formation, and increased cell apoptosis. DBH-AS1 enhanced tumor growth and activated FAK/Src/ERK axis by modulating the expression of miR-138 (Bao et al., 2018). H19 is another up-regulated lncRNA in melanoma. H19 silencing has increased the sensitivity of melanoma cells to cisplatin, suppressed colony formation, and enhanced apoptosis of cisplatin-resistant melanoma cells. This lncRNA regulates IGF-1 expression through modulation of miR-18b expression (An et al., 2020). Honda et al. have assessed the methylation pattern of the H19 differentially methylated region (DMR), loss of heterozygosity, and allelic expression of IGF2 in hepatoblastoma. They reported associations between biallelic IGF2 expression and hypermethylation of H19 DMR. On the other hand, the monoallelic expression of IGF2 has been correlated with normal methylation of this region. They also reported over-expression of IGF2 and predominance of the embryonic P3 transcript in most hepatoblastoma with retention of imprinting (Honda et al., 2008). Table 2 summarizes the role of IGF-associated lncRNAs in cancers.

**TABLE 2:** IGF-associated lncRNAs in cancers (NNTs: nearby normal tissues).

Type of Cancer	lncRNA	*P*-value	Animal	Clinical Samples (Human)	Cell Lines	Target	Pathway	Function	References
NSCLC	circ_0014130	<0.01	–	20 pairs of NSCLC and NNTs	H1299, A549, BEAS-2B	miR-142-5p, IGF-1	–	Downregulation of circ_0014130 via upregulating miR-142-5p and downregulating IGF-1 expression could inhibit NSCLC cell proliferation and promotion of cell apoptosis.	Wang M. et al., 2020
NSCLC	HOXA-AS2	<0.01	–	63 pairs of NSCLC and NNTs	SPCA1, A549, PC-9, H1975, 16HBE	IGF-2	–	HOXA-AS2 via upregulating IGF-2 could promote cell migration and invasion in NSCLC.	Zheng et al., 2019
Cervical Cancer (CC)	LINC00319	<0.01	–	60 pairs of CC and NNTs	Ect1/E6E7, HeLa, SiHa, Caski, C33A, Me180	IGF-1R, miR-147a, Vimentin, MMP2, p21, E-cadherin	–	Downregulation of Linc00319 via targeting the miR-147a and IGF-1R could inhibit CC cell proliferation, invasion, and migration.	Ma et al., 2020
Melanoma	DBH-AS1	<0.001	–	62 pairs of M and NNTs	A375, HaCaT, A875	IGF-1R, miR-223-3p, EGFR, GLUT1	AKT	Overexpression of DBH-AS1 via miR-223-3p/EGFR/AKT axis could enhance the glycolytic activity and reduce cancer progression.	Bao et al., 2018
Melanoma	H19	<0.01	–	30 pairs of M and NNTs	HEMa, A375, WM35, M8, SK-MEL-2, A2508	miR-18b, IGF1	–	H19 via acting as a molecular sponge of miR-18b can regulate IGF-1 expression and sensitivity of melanoma cells to DDP.	An et al., 2020
Hepatoblastoma	H19	<0.05	–	HB (*n* = 54), normal controls (*n* = 5)	HuH6, HepG2	IGF-2, PLAG1, CTNNB1	–	There was an association between biallelic IGF2 expression and hypermethylation of H19 DMR.	Honda et al., 2008
Follicular Thyroid Cancer (FTC)	H19	<0.05	Male nude mice	45 pairs of FTC and NNTs	FTC-133, FTC-238, Nthy-ori 3-1	IGF2BP1, IGF-1, SOCS3, Pax5, miR-29-3p	JAK/STAT	H19 via the IGF1/JAK/STAT axis could suppress metastasis of FTC.	Xu et al., 2019
Breast Cancer (BCa)	IRAIN	<0.05	–	–	MDA-MB-31	IGF-1R	–	IRAIN via targeting IGF1R could alter the phenotypes of MDA-MB-231 tumor cells.	Pian et al., 2018
Bladder Cancer	circVANGL1	<0.001	BALB/c nude mice	60 pairs of BLC and NNTs	SV−HUC, T24, EJ, J82, RT−4, UM−UC−3, TCC	miR−1184, IGFBP2	–	Downregulation of circVANGL1 via inhibiting IGFBP−2 could inhibit cell invasion, migration, and growth.	Yang D. et al., 2020
Breast cancer (BCa)	NR2F1-AS1	<0.0001	Female NOD/SCID mice	–	MDA-MB-231, MCF-7, HUVECs	IGF-1	ERK	Overexpression of NR2F1-AS1 by increasing miR-338-3p and activating IGF-1and ERK pathway could enhance the HUVEC proliferation, tube formation, and migration ability in BCa cells.	Zhang et al., 2020
BCa	SNHG7	<0.05	–	TCGA database	MCF7, T47D, MDA-MB-231, MCF10A	IGF-1	MAPK	The silencing of SNHG7 could lead to cell cycle arrest in G0/G1. A negative feedback loop between SNHG7 and IGF-1 could regulate transcript levels and proliferation in BCa cells.	Boone et al., 2019
Colorectal Cancer (CRC)	circRUNX1	<0.001	Male BALB/c nude mice	52 pairs of CRC and NNTs	SW480, SW620, HCT116, HT29, LoVo, RKO	miR-145-5p, IGF-1	–	Overexpression of circRUNX1 via miR-145-5p/IGF-1 Signaling could enhance cell proliferation and migration and also inhibit apoptosis and metastasis in CRC cells.	Chen et al., 2020
CRC	LINRIS	<0.01	female BALB/c nude mice	60 pairs of CRC and NNTs	LOVO, CCD841, RKO, CW2, SW1116, SW480, SW620, DLD-1, HCT116, HT29, COLO205	IGF2BP2,	–	LINRIS by stabilizing IGF2BP2 could promote the aerobic glycolysis in CRC cells.	Wang et al., 2019e
Colon Cancer	NEAT1	<0.001	–	10 pairs of CRC and NNTs	SW620 HT-29, HCT 116, LoVo, SW480, NCM460	IGF-2, miR-185-5p	–	Overexpressed NEAT1 via the miR-185-5p/IGF-2 axis could promote invasion and migration of colon cancer cells.	Zhuang et al., 2020
Pancreatic Cancer (PaC)	AFAP1-AS1	<0.01		63 pairs of PaC and NNTs	AsPC-1, BxPC-3, PANC-1, PaCa-2, SW1990, HPDE6c7	IGF-1R	–	Downregulation of AFAP1-AS1 by upregulating the IGF1R oncogene via sequestration of miR-133a could suppress the tumor cell growth and invasion in PaC.	Chen B. et al., 2018
PaC	HOTAIR	<0.05	BALB/c nude mice	25 pairs of PaC and NNTs	BXPC3, 293T, CFPAC-1, Panc-1, L3.6pl	IGF-2, miR-663b, Caspase-3, Caspase-9	–	HOTAIR by inhibiting miR-663b via upregulating IFG-2 could promote PaC cell proliferation.	Cai et al., 2016
Renal Cell Carcinoma (RCC)	HOTTIP	<0.01	–	TCGA database, 57 pairs of RCC and NNTs	A-498, 786-O, Caki-1, Caki-2, ACHN, HK-2, 293T	IGF-2, hsa-miR-615-3p	–	HOTTIP by regulating the miR-615/IGF-2 axis could promote RCC progression.	Wang Q. et al., 2018
Glioma	linc01023	<0.001	nude mice	Glioma (*n* = 169), normal brain tissues (NBTs, *n* = 30)	U87, U251, NHA	IGF-1R	AKT	Knockdown of linc01023 by regulating the IGF-1R/AKT axis could restrain glioma proliferation, migration, and invasion.	Yu et al., 2019
Hepatocellular carcinoma (HCC)	DLEU1	<0.01	Male BALB/c nude mice	56 pairs of HCC and NNTs	SMMC-7721, Hep3B, HepG2, Huh-7, LO2	IGF-1R, miR-133a, E-cadherin, N-cadherin Vimentin	PI3K/AKT	DLEU1 by sponging miR−133a to regulate IGF−1R expression through the PI3K/AKT pathway could promote HCC progression.	Zhang W. et al., 2019
Tongue Squamous Cell Carcinoma (TSCC)	THOR	<0.05	–	55 TSCC and 31 NNTs	Tca-8113 and Cal-27	IGF2BP1, IGF-2, Cyclin-E1, Cyclin-D1, p21, p27,	MEK-ERK	THOR by stabilizing IGF2BP1 could increase TSCC cell proliferation.	Wang et al., 2019a

**FIGURE 3:**
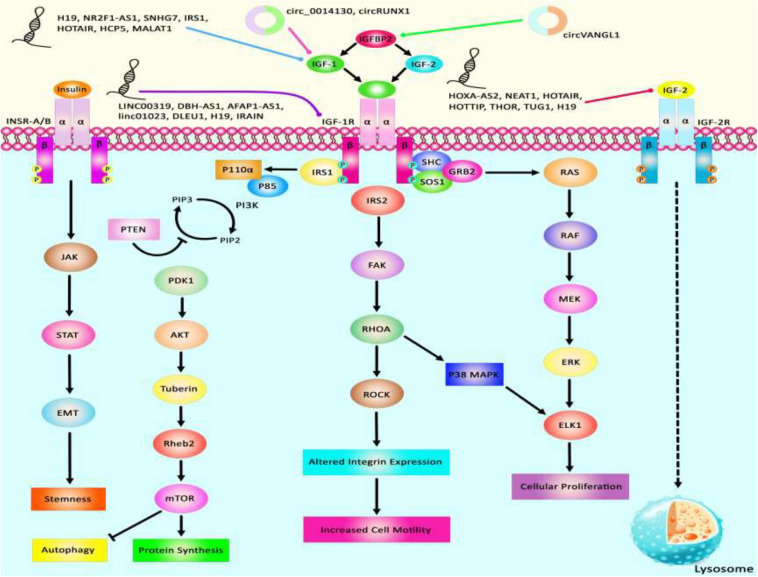
A schematic summary of lncRNAs that target IGF signaling cascade. IGF1, IGF2, IGFBP2, and IGF-1R are among proteins that are regulated by lncRNAs. Abnormal levels of lncRNAs can affect the carcinogenesis process by influencing autophagy, cell proliferation, protein synthesis, and stemness.

Via regulation of the IGF−1 signaling pathway, H19 can modulate proliferation and apoptosis of male germline stem cells. H19 silencing has reduced the cell quantities in the seminiferous tubule (Lei et al., 2019). Expression of the lncRNA 150Rik has been enhanced in renal tissue of animal models of diabetic nephropathy and in mesangial cells cultured in hyperglycemic media. This lncRNA regulates mesangial cell proliferation through interacting with miR-451, thus regulating the IGF1R/p38MAPK axis (Zhang et al., 2018). LncIRS1 has been shown to act as a molecular sponge for miR−15a, miR−15b−5p, and miR−15c−5p to modulate the expression of IRS1 a downstream target of the IGF1-R. Up-regulation of lncIRS1 has enhanced IRS1 expression and increased phosphorylation of AKT as an important element in the IGF-1 pathway. LncIRS1 can also regulate the expression of atrophy−associated genes and affect muscle atrophy (Li Z. et al., 2019). TUG1 is an up-regulated lncRNA in ox-LDL-exposed vascular smooth muscle cell (VSMC) and HUVEC. Its silencing has suppressed proliferation and enhanced apoptosis in ox-LDL-exposed VSMC but has exerted opposite effects in HUVEC. miR-148b has been identified as a target of TUG1 in these cells. In turn, miR-148b has been shown to target IGF2. Therefore, TUG1 enhances IGF2 levels by sequestering miR-148b (Wu X. et al., 2020). HCP5 is a lncRNA that is involved in the pathogenesis of polycystic ovarian syndrome (PCOS). Down-regulation of this lncRNA inhibits cell proliferation via inducing cell cycle arrest at the G1 phase and stimulating the mitochondrial apoptotic route. miR-27a-3p has been recognized as a direct target of HCP5. This miRNA can bind with IGF-1. Therefore, HCP5 can be involved in the development of PCOS via modulating the miR-27a-3p/IGF-1 axis (Luo L.-H. et al., 2020). Figure 3 represents the dysregulation of various types of lncRNAs which have a remarkable role in negatively modulating IGF1, IGF2, IGFBP2, and IGF-1R through the IGF signaling pathway in different human cancers. Table 3 summarizes the information about the role of IGF-associated lncRNAs in non-neoplastic conditions.

**TABLE 3:** IGF-associated lncRNAs in non-malignant disorders.

Type of disorder	lncRNA	*P*-value	Animal	Clinical Samples (Human)	Cell Lines	Target	Pathway	Function	References
–	H19	<0.05	Bovin	–	GC1, mGSCs	IGF-1, IGF-1R, C-MYC, PNCA, p53, Caspase-3,	AKT, ERK	H19 via the IGF−1 pathway could regulate the proliferation of bovine male germline stem cells.	Lei et al., 2019
Diabetic Nephropathy (DN)	150Rik	<0.01	Male db/db mice	–	–	miR-451/IGF1R, Ago2	p38 MAPK	Overexpression of 150Rik via miR-451/IGF-1R/p38 MAPK pathway could promote mesangial cell proliferation in DN.	Zhang et al., 2018
Skeletal Muscle Atrophy	IRS1	<0.05	Hypertrophic (WRR) and leaner broilers (XH)	–	DF-1	IGF1	PI3K/AKT	Overexpression of lncIRS1 via increasing miR-15 to activate IGF1-PI3K/AKT signaling could promote muscle proliferation, differentiation, and muscle mass.	Li Z. et al., 2019
Atherosclerosis	TUG1	<0.001	–	–	HUVEC, VSMC	IGF-2, miR-148b, Bax, Bcl-2, PCNA	–	TUG1 by regulating the miR-148b/IGF2 axis could regulate apoptosis and proliferation in ox-LDL-stimulated HUVEC and VSMC.	Wu X. et al., 2020
Polycystic Ovary Syndrome (PCOS)	HOTAIR	<0.01	Rat	–	Granulosa cells	IGF-1, miR-130a	–	Downregulation of HOTAIR by reducing the expression of IGF-1 via miR-130a could alleviate PCOS in rats.	Jiang et al., 2020
Polycystic Ovarian Syndrome (PCOS)	HCP5	<0.001	–	–	KGN	IGF-1, miR-27a-3p, caspase-9, Bax, Bcl-2	–	Downregulation of HCP5 via the IGF-1/miR-27a-3p axis could induce apoptosis and also could suppress cell proliferation by arresting cell cycle progression at the G1 phase.	Luo L.-H. et al., 2020
Preeclampsia (PE)	MALAT1	<0.01	–	30 pairs of PE and matched normal pregnant women	HTR-8/SVneoc, JEG-3	miR-206, IGF-1	PI3K/AKT	Downregulation of MALAT1 via knockdown of IGF-1 and upregulating miR-206 could suppress the trophoblast cell migration and invasion.	Wu H. Y. et al., 2020
PE	MALAT1	<0.01	–	30 patients with PE and 30 normal samples	HTR-8/SVneoc, JEG-3	IGF-1, miR-206	PI3K/AKT	MALAT1 via regulating the miR-206/IGF-1 axis through the PI3K/AKT pathway could regulate trophoblast cell migration and invasion.	Wu H. Y. et al., 2020
Liver Fibrosis	H19	<0.01	Male Sprague-Dawley rats	–	HSC-T6	IGF1R, MeCP2, a-SMA, Col1A1, miR-200a	–	H19 by targeting the MeCP2/IGF1R axis control hepatic stellate cell proliferation.	Yang et al., 2016
Endometriosis	H19	=0.035	–	–	Human endometrial stromal cells	IGF-1R, let-7	–	Downregulation of H19 via the let-7/IGF-1R axis could reduce the proliferation of endometrial stromal cells.	Ghazal et al., 2015

LncRNAs that regulate the expression of IGF1, IGF2, IGF-1R, and IGFBPs can participate in the pathogenesis of human disorders. H19, NR2F1-AS1, and SNHG7 participate in the development of melanoma and breast cancer through modulation of IGF1 (Boone et al., 2019; An et al., 2020; Zhang et al., 2020). NEAT1, THOR, and HOTTIP via targeting IGF2 affect carcinogenic processes in colorectal cancer, tongue squamous cell carcinoma, and renal cell carcinoma (Wang Q. et al., 2018; Yang et al., 2019; Zhuang et al., 2020). Additionally, downregulation of circVANGL1 through suppressing the expression level of IGFBP2 could attenuate breast cancer cell invasion, migration, and proliferation (Yang D. et al., 2020). Also, IRAIN, Linc00319, and DLEU1 through negatively regulating IGF-1R could cause breast cancer, cervical cancer, and hepatocellular carcinoma (Pian et al., 2018; Zhang W. et al., 2019; Ma et al., 2020). Figure 3 summarizes the role of these IGF-associated lncRNAs in human disorders.

## Diagnostic/Prognostic Values of IGF-Associated miRNAs/lncRNAs in Cancers

A number of miRNAs and lncRNAs which are functionally linked with IGF signaling have potential applications as diagnostic/prognostic markers in cancers. Zhuang et al. have demonstrated high accuracy of NEAT1 levels in distinguishing colon cancer tissues from normal ones (area under the receiver operating curve = 0.89) (Zhuang et al., 2020). Expression levels of the IGF-associated miRNAs miR-485-5p and miR-155-5p have been associated with the survival of patients with lung cancer and Wilms tumor, respectively (Huang et al., 2018; Luo X. et al., 2020). Also, Linc00319, H19, AFAP1-AS1, SNHG7, HOTTIP, linc01023, DLEU1, and NEAT1 have been identified as prognostic markers in diverse kinds of cancer (Table 4).

**TABLE 4:** Prognostic values of miRNAs/lncRNAs in cancers (NNTs: nearby normal tissues).

Sample number	Kaplan-Meier analysis	Univariate/multivariate cox regression	References
87 pairs of lung cancer and NNTs	Downregulation of miR-485-5p was correlated with poor prognosis in NSCLC.	–	Huang et al., 2018
87 pairs of WT tissues and paracarcinoma kidney tissues	miR-155-5p and IGF2 level did not correlate with the survival time of WT patients.	–	Luo X. et al., 2020
60 pairs of cervical cancer samples and NNTs	Higher Linc00319 expression level was related to the low survival rate	–	Ma et al., 2020
44 pairs of resected OSCC and NNTs	Downregulation of miR-375 is correlated with tumor progression and poor prognosis of OSCC patients.	–	Zhang et al., 2017
60 pairs of HCC tissues and NNTs	Reduced expression of miR-505 was correlated with the worse prognosis of HCC patients.	Tumor size, Lymph node metastasis, and TNM stage were correlated with prognosis.	Ren et al., 2019
150 pairs of HCC tissues and NNTs	Downregulation of miR-216b was correlated with poor prognosis in HCC.	–	Liu F. Y. et al., 2015
62 paired HCC samples and NNTs	Downregulation of miR-29a-3p was correlated with poor prognosis in HCC.	–	Wang X. et al., 2017
80 pairs of CRC and NNTs	The decreased miR-491-5p expression level was associated with poor overall survival in CRC patients.	Differentiation level and TNM stage were correlated with prognosis.	Lu et al., 2019
30 pairs of malignant melanoma tissues and NNTs	Lower expression of lncRNA H19 was associated with better overall survival.	–	Men et al., 2020
89 pairs of LGG tissues and NNTs	Low expression of miR-138 was associated with poor prognosis in LGG patients.	–	Yang Y. et al., 2020
90 pairs of GC tissues and NNTs	Downregulation of miR-598 was correlated with poor prognosis in GC.	–	Liu et al., 2018c
63 pairs of PC and NNTs	Upregulation of AFAP1-AS1 was correlated with poor overall survival in patients with PC.	–	Chen B. et al., 2018
40 pairs of glioblastoma tissues and non-tumor tissues	Lower expression of miR-15b was associated with a shorter survival rate.	–	Wang J. et al., 2017
TCGA database	Higher expression of SNHG7 was associated with a lower OS rate.	–	Boone et al., 2019
TCGA database, 57 pairs of RCC and NNTs	Higher expression of HOTTIP was associated with lower OS and DFS rates.	Higher expression of HOTTIP was associated with pathological grade, tumor size, and TNM stage.	Wang Q. et al., 2018
Glioma (*n* = 169), NBTs (*n* = 30)	Higher expression of linc01023 was associated with a lower OS rate.	–	Yu et al., 2019
56 pairs of HCC and NNTs	Higher expression of DLEU1 was associated with a lower OS rate.	–	Zhang W. et al., 2019
10 pairs of CRC and NNTs	Higher expression of NEAT1 was associated with a lower OS rate.	–	Zhuang et al., 2020

## Importance of IGF-Associated Pathways in Response to Chemotherapy

IGF-associated molecules have been involved in the resistance of cancer cells to chemotherapeutic agents. In some cases, miRNAs or lncRNAs have been identified as molecules that mediate this phenotype. For instance, H19 silencing has enhanced the sensitivity of cancer cells to cisplatin and increased apoptosis of cisplatin-resistant melanoma cells through modulation of IGF1 expression (An et al., 2020). In a number of ovarian cancer cell lines, IGF-2 expression has been higher in Taxol-resistant cells compared with chemosensitive cell lines. Transient IGF2 silencing has enhanced Taxol sensitivity in these cells. However, IGF1R blocking did not affect the chemosensitivity of these cells. These results have supported the role of IGF-2 as a possible therapeutic target in drug-resistant ovarian cancer (Brouwer-Visser et al., 2014). IGF-1 has been shown to confer resistance to docetaxel in prostate cancer cells. IGF-I treatment has reduced expression of miR-143 expression, while enhanced expression IGF-1R and IRS1, direct targets of this miRNA. Up-regulation of miR-143 has stopped IGF-I-associated resistance to docetaxel, reduced expressions of IGF-I, IRS1, and VEGF in these cells (Niu et al., 2017). Table 5 reviews the importance of IGF-related pathways in response to chemotherapy.

**TABLE 5:** Importance of IGF-associated pathways in response to chemotherapy.

Type of cancer or disease	microRNA/lncRNA	Animal	Clinical Samples (Human)	Cell lines	Target	Pathway	Function	References
Melanoma	H19	–	30 pairs of M and NNTs	HEMa, A375, WM35, M8, SK-MEL-2, A2508	miR-18b, IGF1	–	Downregulation of H19 could increase the sensitivity of melanoma cells to DDP.	An et al., 2020
Ovarian cancer (OC)	–	Female athymic nude mice (Harlan)	489 cases of high grade serous OC	A2780, HEY, NIH: OVCAR-8, HET-T30, A2780-T15, HEY-B20, HEY-Epo8, OVCAR-8-D30	IGF-2	AKT, ERK	Downregulation of IGF-2 could activate taxol sensitivity in drug-resistant OC.	Brouwer-Visser et al., 2014
OC	–	–	–	HEY, OVCAR-8, SKOV-3, BG-1, A2780, HEY-T30	IGF-1R	PI3K, ERK	Overexpression of the IGF-1 could induce cisplatin resistance of OC cells.	Eckstein et al., 2009
OC	–	–	134 pairs of OC and NNTs	A2780, HEY, HEY-T30	IGF2	AKT	Downregulation of IGF-2 could reduce drug-resistant OC cells to taxol.	Huang et al., 2010
OC	–	–	212 pairs of OC and NNTs	–	IGF-II	–	IGF-II and its SNP could be associated with elevated risks of disease progression and death in epithelial OC.	Lu et al., 2013
Breast Cancer (BCa)	–	–	–	Hs578T, Hs578T/PTX	IGFBP-3, Caspase-3	–	Paclitaxel could increase the endogenous IGFBP-3 production, then induce apoptosis of Hs578T human BCa cells.	Fowler et al., 2000
BCa	–	–	–	MCF-7, CAMA-1, MDA-MB-361, HCC1954, BT474, MDA-MB-453, UACC893, HCC70, MDA-MB-435S, ZR75-30, HCC1419, SKBR-3, BT549, T47D, ZR75-1, MDA-MB-231	IGF-1R, IRS-1	PI3K/AKT	NVPAEW541 treatment via Inhibiting IGF-1R and the PI3K/AKT pathway could lead to inhibit cell growth and increase the effect of chemotherapeutic drugs.	Mukohara et al., 2009
BCa	–	–	24 BC patients and 16 healthy women	–	IGF-1	–	Adjuvant cyclophosphamide, methotrexate, and 5-fluorouracil could decrease the plasma concentration of IGF-1 in premenopausal BCa women.	Kajdaniuk et al., 2001
BCa	–	–	73 pairs of BCa and NNTs	Py230, 4T1, MDA-MB231	IGF-1/2	–	Downregulation of IGF1/2 in combination with paclitaxel could reduce tumor cell proliferation and lung metastasis in pre-clinical BCa models.	Ireland et al., 2018
Triple-Negative Breast Cancer (TNBC)	–	Female BALB/c nude	–	MDA-MB-468, HCC1806	GFBP-3, EGFR, S1P, SphK-1, Caspase-3	–	Inhibition of EGFR and SphK could lead to a block of IGFBP-3-dependent signaling and inhibit cell proliferation in TNBC.	Julovi et al., 2018
Pancreatic Cancer	–	Female Fox Chase SCID Beige mice	–	KP-4, BxPC-3, Capan-2, CFPAC-1, HPAF-II, SU8686, SW1990, AsPC1, PANC1	IGF-1R, ErbB3	PI3K/AKT	IGF-1 and HRG by targeting the PI3K/AKT pathway could reduce pancreatic cancer cell sensitivity to gemcitabine or paclitaxel.	Camblin et al., 2018
Prostate Cancer	miR-143	Male BALB/cA-nu nude mice	–	PC-3, DU145, 293T, DU145/DTX, PC-3/DTX	IGF-1/R, VEGF	–	Overexpression of miR-143 could reduce IGF-1-induced chemoresistance to docetaxel treatment and inhibit tumor growth *in vivo*.	Niu et al., 2017
Prostate Adenocarcinoma (PaC)	–	–	–	DU145, PC-3, DU145/DTX, PC-3/DTX	IGF-I, IGFBP-2	PTEN	Downregulation of IGFBP-2 could increase the sensitivity of Cap cells to docetaxel.	Uzoh et al., 2011
CRC	miR-143	–	62 pairs of CRC and NNTs	–	IGF-1R	–	Upregulation of miR-143 by targeting IGF-1R could inhibit cell proliferation, migration, and also increase chemo-sensitivity to oxaliplatin.	Qian et al., 2013
CRC	–	–	–	WiDr, SW620, HMEC-1,	IGF-1, HIF-1α	–	IGF-1 could increase the cell viability of stromal and cancer cells in response to chemotherapy in CRC.	Volkova et al., 2014
CRC	miR-497		131 pairs of CRC and NNTs	HCT116, HCT28, LoVo, Colon205, SW480, SW620, CRL-1831	IGF-1R	PI3 K/AKT	Overexpression of miR-497 via inhibiting IGF1-R activity could increase sensitivity to apoptosis induced by chemotherapeutic drugs in CRC cells.	Guo et al., 2013
Adrenocortical Carcinoma (ACC)	–	–	17 ACCs and 6 normal adrenal tissue samples samples	H295R, HAC15	IGF-2, IRA, IGF-1R, IGF-2R	mTOR	Linsitinib treatment by IGF pathway could inhibit cell growth in the H295R and HAC15 cell lines	De Martino et al., 2019
Gastric Cancer	–	–	3 pairs of GC and NNTs	–	IGFBP-3, ICAM-1, VCAM-1, p65, NF-kB, IkB	–	Overexpression of IGFBP-3 could increase cell growth inhibition via suppressing the NF-kB activity by regulating ICAM-1 and VCAM-1 in GC cells.	Kim and Lee, 2015
Gastrointestinal Stromal Tumor (GIST)	–	–	–	GIST-882, GIST-T1, GIST-882/imatinib, GIST-T1/imatinib	IGF-1R,	–	Knockdown of CCDC26 by regulating IGF-1R could induce imatinib resistance in GIST cells.	Xie et al., 2019
Esophageal Squamous Cell Carcinoma (ESCC)	–	–	–	SLMT-1, SLMT-1/CDDP1R, SLMT-1R-pcMV3, SLMT-1R-IGFBP5	IGFBP5	–	Downregulation of IGFBP5 could induce cisplatin-resistance in ESCC cells.	Chan et al., 2018
Brain Tumor	–	–	–	MCH-BT-31, MCH-BT-39, MCH-BT-30, MCH-BT-52, HTB-14	IGF-1R, IGF-I, IGF-II, P-gp	PKC	Tamoxifen treatment could reduce PKC activity and IGF-II expression in brain tumor cells.	Ramachandran et al., 2004
Glioma	–	–	–	U-87MG, KNS-42	CPP32, Bcl-2	p53	Overexpression of IGF-I by increasing the expression of Bcl-2 and decreasing the activity of CPP32 could decrease apoptosis in glioma cells.	Yin et al., 2005
Non-Small-Cell Lung Carcinoma (NSCLC)	–	Nude mice	NSLC (*n* = 14), patients without cancer (*n* = 9)	SCC-25, HeLa, SCC-25/CP, KB-3-1, KB-CP, 2008/CP, IGROV1/CP, A2780/CP, PC-9/CDDP, PC-14/CDDP, SBC-3/CDDP	IGFBP7, MKP3	MAPK Erk1/2, MEK/Erk, Stat3	Downregulation of IGFBP7 could increase cellular resistance to cisplatin.	Okamura et al., 2012
NSCLC	–	–	–	SKMES1, SKMES, SKLu-1, Calu-3, H1299, H460, H157, HCC44, A549, H1975	IGF-1, IGF-1R, VEGF	AKT, MAPK	AVE1642 treatment by targeting IGF-1 could increase the paclitaxel-mediated anti-tumor effect.	Spiliotaki et al., 2011
NSCLC	miR-223	Male nude mice	–	PC-9, PC-9/ER, PC-9/ER-EV	IGF-1R	AKT/S6	Overexpression of miR-223 by relating IGF-1R could inhibit tumor growth in nude mice and also increase the sensitivity to erlotinib.	Zhao et al., 2016
NSCLC	–	–	–	NCI-H460, H1299, A549	IGF-1, Chk2, Chk1	p53	Overexpression of IGF-1 could recover cisplatin-derived inhibition of proliferation and apoptosis in NSCLC cells.	Jeon et al., 2008
NSCLC	–	Female athymic nude mice	–	H460	IGF-1, IGFBP-3	–	Targeting of the IGF-1 receptor using siRNA could result in the sensitization of cisplatin-R-cells to cisplatin and radiation.	Sun et al., 2012
NSCLC	–	–	–	A549, A549/PTX	IGF-1, SphK1, Vimentin, Fibronectin, N-cadherin, E-cadherin	ERK, AKT	IGF-1 treatment via activating SphK1, ERK, and AKT could decrease the sensitivity of A549 cells to paclitaxel.	Wu et al., 2019
NSCLC	LUCAT1	–	–	A549/DDP, A549	IGF-2	–	Overexpression of LUCAT1 by regulating IGF-2 could promote the cisplatin resistance in NSCLC.	Wang et al., 2019c
Cardiac Toxicity	–	–	–	H9c2	IGF-1R, IGFBP-3	p53	Doxorubicin via inhibiting IGF-1R and upregulating IGFBP-3 through p53 could lead to resistance to IGF-1 that may contribute to doxorubicin-initiated apoptosis.	Fabbi et al., 2015

## IGF Signaling Pathway in Tumorigenesis and Progression of Chemotherapeutic Drug Resistance Providing the New Concepts in Cancer Therapy

One of the major impediments to current cancer remedy endeavors is the induction of drug resistance by tumors. Despite recent improvements in diagnostic methods and surgical interventions, many aggressive tumors have a poor response to adjuvant or neoadjuvant chemotherapy and radiation. The IGF signaling axis has been detected to have a pivotal role in the progression and development of a variety of tumors (Denduluri et al., 2015). The IGF-1R is involved in various human cancers, such as ovarian, breast, pancreatic, glioma, hepatocellular, lymphoma, and non-small lung cancers. In some cases, its anti-apoptotic attributes strengthen cancerous cells to resist the cytotoxic characteristics of chemotherapeutic agents or radiotherapy (Beauchamp et al., 2009; Dool et al., 2011; Awasthi et al., 2012; Zhou, 2015). Zhou et al. demonstrated that the IGF-1R kinase inhibitor nVp-ADW742 combined with temozolomide could trigger inhibition of P38, GSK3β, and AKT phosphorylation along with a considerable reduction in the intracellular expression levels of Bcl-2, P38, and GSK3β, thereby resulting in promoting response to chemotherapeutic drug temozolomide in medulloblastoma to a large extent (Zhou et al., 2011). Also, Vewinger et al. have illustrated that the IGF signaling pathway has an important role in HGNET-BCOR brain tumor since IGF-1R could be a significant target to improve the sensitivity of vinca alkaloids, vinblastine, doxorubicin, ceritinib, and actinomycin D as efficient drugs in patients affected with this kind of brain tumor. As a consequence, utilizing the off-target IGF1R suppressor ceritinib may pave the way for the remedy of tumor cells driven by IGF1R and IGF2 (Vewinger et al., 2019). In another study, Valerie et al. have indicated that the activity of histone deacetylase inhibitors (HDACi) has reduced in Ewing sarcoma patients. Drug combinations of temozolomide with the dual ALK and IGF-1R inhibitor, AZD3463 could suppress AKT and STAT3 to promote the cytotoxic impacts of temozolomide, and thereby decreasing cell proliferation and enhancing apoptosis via cleavage of PARP and caspase-3 indicating that AKT and STAT3 activation could be modulated by ALK and IGF-1R signaling pathway (Sampson et al., 2015). Additionally, Refolo et al. have figured out that the combined treatment with regorafenib, vitamin K1, and two IGF-1R tyrosine kinase inhibitors GSK1838705A or OSI-906 could strengthen antitumor effects of the target drug, improving their actions and decreasing their toxicity to a large extent. Therefore, both IGF1-R inhibitors could enhance the pro-apoptotic and antiproliferative impacts of regorafenib and VK1 in hepatocellular carcinoma downregulating both MAPK and PI3K/AKT signaling pathways (Refolo et al., 2017). Supplementary Table 2 summarizes the results of various studies that indicate utilizing IGF-1R drug inhibitors with the aim of suppressing the anti-apoptotic properties of IGFR which cause cancerous cells to resist the cytotoxicproperties of chemotherapeutic drugs or radiotherapy.

## Epigenetic Regulation of IGF-I, IGF-II, IGF-1R, and IGFBPS of IGF Axis in a Variety of Human Cancers

Accumulating evidence indicates that dysregulation of epigenetic systems has an important role in cancer pathogenesis resulting in overexpression of altered target genes as well as malignant cellular transformation. Since the IGF axis could contribute to cancer progression and invasion, it is now widely accepted that aberrant methylation of IGFBP7, IGFBP-4, IGFBP-3, IGF-1R, IGF-1, and IGF-II promoters could be a potential factor in various common human cancers (Qian et al., 2011; Sato et al., 2011; Bolomsky et al., 2015; Ye P. et al., 2016). Beeghly et.al have demonstrated that differential promoter P2 and P3 methylation patterns of the IGF-II gene could be remarkably related to promoting the risk of disease progression in epithelial ovarian cancer, especially hypermethylation of P2 could be associated with unpleasant symptoms of this serious disease (Beeghly et al., 2007). Additionally, another research indicated that epigenetic alterations in the IGF signaling pathway could play an effective role in the emergence of hepatocellular carcinoma. Therefore, considerable demethylation and upregulation of IGFBP3 via employing 5-Aza-2′- deoxycytidine and trichostatin A therapy results in attenuating cell proliferation and decreasing colony formation in HCC cells (Han et al., 2015). Chang et al. have illustrated that hypermethylation of the IGFBP-3 promoter which dramatically suppressed the expression level of this target gene could be substantially related to poor prognosis among Non-Small Cell Lung Carcinoma patients. Therefore, utilizing demethylation agents to upregulate the expression of IGFBP-3 could pave the way for providing a pivotal remedial procedure for these patients (Chang et al., 2002). Besides, Dar et al. have discovered that epigenetic silencing of IGFBP3 via hypermethylation of its promoter in human melanoma cells. Upregulation of IGFBP3 through applying 5AZA treatment resulting in inhibiting cancer cell survival, triggering tumor cell death, decrease colony formation and invasion, inducing expression of the pro-apoptotic genes containing PUMA, p21, and BAX as well as caspase 3 cleavage and downregulating phosphorylation of AKT (Dar et al., 2010). Furthermore, Schayek et al. have indicated that hypermethylation of AR promoter in metastatic prostate cancer cells results in downregulation of IGF1R expression levels which indicates the fact that the IGF1R gene has been detected s a downstream target for AR action. Employing 5-Aza treatment could trigger demethylation of AR promoter and as a consequence the expression level of IGF1R could increase significantly which may consider as a promising therapy in human prostate cancer (Schayek et al., 2010). An overview of promoter methylation and epigenetic modulation of various genes relevant to the IGF signaling pathway in different human cancers is represented in Supplementary Table 3.

## Applying Remedial Crispr and siRNA State-Of-The-Art Genome Editing Systems to Manipulate the IGF Signaling Pathway in Various Human Cancers

It is now accepted that gene silencing via CRISPR-Cas9 and small interfering RNA (siRNA) is becoming an inevitable gene-editing tool in biological research, especially to repair genetic defects via editing or knock out various genes related to the IGF signaling pathway. Via applying a CRISPR/Cas9 or siRNA genome editing tool, it could be possible to knock out or edit ectopic expression of various genes related to IGF signaling cascade through which we could be able to improve response to chemotherapeutic agents as well as attenuating tumor cell survival, proliferation, invasion, angiogenesis, and metastasis of different kinds to a large extent (Singh et al., 2008; Brouwer-Visser et al., 2014; Hussmann et al., 2017; Strub et al., 2018). Liu et al. have detected that knockdown of IGF2BP1 expression level through applying a CRISPR/Cas9 genome editing system could play a crucial role in repressing the expression levels of IGF2, Gli1, CD44, and Myc in skin SCC cells through which tumor cell proliferation and survival were suppressed considerably. Likewise, via utilizing siRNA-mediated knockout of IGF2BP1-bound lncRNA THOR, skin SCC cell growth could be suppressed dramatically (Liu et al., 2018e). In addition, another research demonstrated that silencing IGF1R expression through employing a CRISPR/Cas9 genome editing system leads to functional endpoint mechanism for TKI resistance in a targetable direction MET-amplification, and thereby resulting in improving response to treatment via suppressing resistance to Erlotinib in Non-Small Cell Lung Carcinoma cells and inhibiting epithelial-mesenchymal transition in tumor cells (Hussmann et al., 2017). Besides, Strub et al. have demonstrated that via applying a CRISPR–Cas9 screen targeting chromatin regulators the histone deacetylase SIRT6 haploinsufficiency could play an effective role in upregulating IGFBP2 expression level through promoting chromatin availability, H3K56 acetylation at the IGFBP2 locus, and overexpression of IGF-1R function as well as downstream AKT signaling cascade. Additionally, elevating the IGFBP2 expression could lead to attenuate sensitivity to MAPK signaling inhibitors, and thereby increasing BRAFV600E melanoma cell survival via triggering IGF-1R/AKT signaling pathway. Thus, incorporating a clinically suitable IGF-1Ri with BRAFi could pave the way for promoting the sensitivity of SIRT6 haploinsufficient melanoma cells (Strub et al., 2018). Besides, another research indicated that POU2F3 can be expressed particularly in variant SCLC cancers that have the insufficient expression of neuroendocrine markers and markers of a chemosensory lineage. They applied domain-focused CRISPR screening as a suitable procedure to identify POU2F3 as a significant transcription factor in a subset of SCLC cells and to display other important associations in POU2F3-expressing SCLC lines, containing the lineage TFs SOX9 and ASCL2 and IGF1R. Besides, this strategy shed light on the fact that upregulation of IGFBP5 through employing lentivirus in POU2F3high SCLC lines could suppress tumor cell growth remarkably (Wu et al., 2018). Baade Rø et al. have illustrated that there are an extreme intricacy and interaction between the chemokine and cytokine network triggering migration. They have detected the positive relevance among the degree of cytokine-induced migration and phosphorylation of PAK. PAK phosphorylation was considerably elevated when tumor cells were triggered by combinations of SDF-1a, IGF-1, and HGF which could play an effective role in promoting myeloma cell migration to the large extent. Therefore, via utilizing small interfering RNA, the expression of PAK was downregulated leading to attenuating cytokine-driven migration (Rø et al., 2013). Another study detected that silencing expression of IGFBP-6 or IGF-I or IGF-II through applying siRNA mechanism as well as knockdown IGF-1R activity on fibroblasts could lead to altering fibroblast mobilization, attenuating tumor invasion and TME remodeling through the IGFs/IGF-1R axis in breast epithelial cells which can be considered as a helpful tool for pivotal therapeutic of breast cancer related to dysregulation of IGF signaling pathway (De Vincenzo et al., 2019). Additionally, Brouwer-Visser et.al indicated that suppressing the expression level of IGF2 in ovarian cancer cells via employing RNA interference technology could elevate paclitaxel sensitivity and could restore sensitivity to both microtubule-stabilizing and destabilizing agents (Brouwer-Visser et al., 2014). A summary of clinical researches with the aim of editing or knocking down aberrant expression of different target genes relevant to IGF signaling pathway in various human cancers via employing CRISPR/Cas9 and siRNA gene-editing tools are demonstrated in Tables 6, 7, respectively.

**TABLE 6:** Pre-clinical studies employing the CRISPR/Cas9 system with the aim of editing or knocking down various target genes related to the IGF signaling pathway in different human cancers.

Type of cancer or disease	Target	*In vitro*	Cell line	Animal	*In vivo*	CRISPR	Vector	Treatment	Pathway	Function	References
Diffuse large B-cell lymphoma (DLBCL)	YAP	+	LY1, LY3	5-week-old female SCID beige mice	+	Knockout	Lentiviral	Doxorubicin	Hippo-YAP	Knockdown of YAP expression level through a CRISPR/Cas9 genome editing system accompanied with suppression of the expression level of IGF-1R leading to the activation of downstream targets CTGF and CYR61, and thereby could remarkably inhibit tumor cell proliferation and cause cell cycle arrest in DLBCL cells.	Zhou et al., 2020
BRAF V600E melanoma	SIRT6	+	SKMel-239	6-week-old female athymic mice (NCrnu/nu)	+	Screening (targeting ∼140 chromatin factors)	Lentiviral	Dabrafenib, Trametinib	IGF-1R/AKT	Employing CRISPRCas9 screen targeting chromatin regulators illuminate that SIRT6 haploinsufficiency could upregulate IGFBP2 expression level as well as attenuate sensitivity to MAPKi, and thereby enhancing BRAFV600E melanoma cell survival via triggering IGF-1R/AKT signaling pathway.	Strub et al., 2018
BRAF V600E melanoma	PTRF	+	MM121224	–	–	Knockout (targeting exon 1)	Lentiviral	Vemurafenib, Encorafenib	TGFβ, MAPK, IGF	Proteomic analysis of CRISPR/Cas derived PTRF knockouts demonstrated that two markers (PTRF and IGFBP7), which are considerably overexpressed, have an effective contribution to MAPKi resistance and EMT as well as promoting cell adhesion and sphere formation in melanoma cells harboring BRAF mutations.	Paulitschke et al., 2019
Skin squamous cell carcinoma (SCC)	IGF2BP1	+	A431	SCID mice	+	Knockout	Lentiviral	–	–	Knockdown of IGF2BP1 expression level via a CRISPR/Cas9 genome editing system could downregulate the expression levels of IGF2, Gli1, CD44, and Myc, and thereby attenuating proliferation and survival of skin SCC cells.	Liu et al., 2018e
Breast cancer (BCa)	IRAIN	+	MDA-MB-231	–	–	Insertion a strong CMV promoter in front of IRAIN to upregulate IRAIN lncRNA via inducing homologous recombination	Lentiviral	–	IGF1R	Via employing CRISPRCas9 gene-editing system IRAIN could compete in cis with the overlapping IGF1R promoter, and thereby suppress the IGF1R signaling cascade that in turn attenuate tumor cell proliferation and metastasis in BCa cells.	Pian et al., 2018
Colorectal cancer (CRC)	CXCR4	+	HT115, COLO201	–	–	Knockout	Plasmid	–	IGF1R	Knockdown of CXCR4 expression level via applying a CRISPR/Cas9 genome editing system in CRC cells could inhibit tumor angiogenesis triggered via IGF1R with the help of SDF-1 in the tumor microenvironment.	Zheng et al., 2017
Ewing sarcoma (EWS)	PAPPA	+	EW8	6–8-week-old male NSG mice	+	Knockout	Not available	–	IGF-1	Knockdown of PAPPA expression level via applying a CRISPR/Cas9 genome editing system could overwhelmingly attenuate immune evasion in EWS cells triggered by PAPPA via reinforcement of IGF-1 signaling.	Heitzeneder et al., 2019
Glioblastoma (GBM)	ERN1, IGFBP3, IGFBP5	+	U251	–	–	Knockout	Plasmid	12ADT	IGF-1, IRE1α	Inhibition of the expression levels of ERN1, IGFBP3, and IGFBP5 via applying a CRISPR/Cas9 genome editing system could promote sensitivity to 12ADT in GBM cells.	Rodvold et al., 2020
GBM	IGF2BP1	+	A172	–	–	Knockout	Lentiviral	–	–	Knockdown of IGF2BP1 expression level via applying a CRISPR/Cas9 genome editing system leading to upregulation of miR-4500 in GBM cells, and thereby suppressing tumor cell growth and metastasis to a large extent.	Li Z.-W. et al., 2019
Liver cancer stem cells (liver CSCs)	β-Catenin	+	Huh7	–	–	Knockout (targeting exon 1 and 5)	Lentiviral	–	Wnt/β-catenin, IGF/MEK/ERK	Inhibition of the expression level of β-catenin via applying a CRISPR/Cas9 genome editing system demonstrated that IGF/MEK/ERK triggers Tcf7l1 phosphorylation and ubiquitination and controlling its suppression independent of β-catenin in liver CSCs.	Shan et al., 2019
Lung cancer (LC)	Nrf2	+	A549	11–12-week-old female C.B-17 SCID.beige mice	+	Knockout	Lentiviral	–	IGF1R	Suppression of the expression level of Nrf2 via applying a CRISPR/Cas9 genome editing system illustrated that ERBB3 and IGF1R signaling pathway accompanied by thioredoxin and peroxiredoxin proteins play an effective role in KEAP1-mutant cancer cells.	Vartanian et al., 2019
Non-Small Cell Lung Carcinoma (NSCLC)	IGF1R	+	HCC827	–	–	Knockout (targeting exon 2 leading to a deletion of 101 bp)	Plasmid	Erlotinib	IGF1R	Knockdown of IGF1R expression level via applying a CRISPR/Cas9 genome editing system could promote the responsiveness of NSCLC cells to Erlotinib, and thereby suppressing EMT.	Hussmann et al., 2017
Oral squamous cell carcinoma (OSCC)	IGF1R	+	SCC-4	–	–	Knockout	Lentiviral	–	PI3K-AKT, hedgehog	Knockdown of IGF-1 expression level via applying a CRISPR/Cas9 genome editing system could suppress the activation of AKT and Hedgehog signaling pathways, and thereby inhibiting cell proliferation, migration, and tumor aggressiveness in OSCC cells.	Ferreira Mendes et al., 2020
Osteosarcoma (OS)	IGF1, IGFBP3	+	U2OS	–	–	Knockout	Not available	Graphene Oxide nanoparticles	IGF1, IGFBP3	Knockdown of IGF1 and IGFBP3 expression level via applying a CRISPR/Cas9 genome editing system could promote apoptosis in OS cells which in turn leading to downregulating the expression level of ROS and Nrf-2, and thereby enhancing the sensitivity of Graphene Oxide in tumor cells.	Burnett et al., 2020
Prostate cancer (PCa)	MDA-9/syntenin	+	ARCaPM	Athymic nude mice	+	Knockout	Plasmid	–	STAT3	Knockdown of MDA-9/syntenin expression level via applying a CRISPR/Cas9 genome editing system could downregulate the expression levels of MMP-2 and MMP-9 and inhibit STAT3 activation as well as suppressing pro-angiogenic factors containing IGFBP-2, VEGF-A, IL-8, and IL-6, and thereby attenuating invasion in PCa cells.	Das et al., 2018
Renal cell carcinoma (RCC)	THOR	+	786-O	5–6-week-old female nude mice	+	Knockout	Plasmid	–	–	Knockdown of THOR expression level via applying a CRISPR/Cas9 genome editing system could suppress the expression levels of IGF2BP1-regulated genes, containing IGF2, Myc, and GLI1, and thereby inhibiting proliferation and viability of RCC cells.	Zhu W. et al., 2018

**TABLE 7:** Pre-clinical researches applying the siRNA silencing mechanism to edit or knockdown aberrant expression of target genes relevant to the IGF signaling pathway in various human cancers.

Type of cancer or disease	Target	*In vitro*	Cell line	Animal	*In vivo*	siRNA	Vector	Treatment	Pathway	Function	References
Breast cancer (BCa)	IGF-1R	+	EMT6, C4HD	BALB/c females	+	Inhibition of IGF-1R expression	Cytomegalovirus (CMV)	–	IGF-1R, AKT, ERK	Attenuating tumor cell proliferation, Suppressing phosphorylation of downstream signaling cascades ERK and AKT, Triggering secretion of proinflammatory cytokines IFN-γ and TNF-α, and blocking G0/G1 cell cycle.	Durfort et al., 2012
BCa	IGF-II	+	MCF-7	–	–	Inhibition of IGF-II expression	Not available	Resveratrol (RSV)	PI3K/AKT, MAPK/ERK	Enhancing progression and chemoresistance in BCa cells via negatively regulating Bcl-2 and Bcl-XL.	Singh et al., 2008
BCa	IGF-1R	+	SKBR3	–	–	Inhibition of IGF-1R expression	Plasmid	Docetaxel	IGF-1R	Utilizing the MUC1 Apt-conjugated CH NPs with the aim of co-delivery of Docetaxel and IGF-1R siRNA remarkably inhibiting the expression levels of IGF-1R, MMP9, STAT3, and VEGF.	Jafari et al., 2019
BCa	IGFBP-6, IGF-I, IGF-II, IGF-1R	+	MCF10A-MycER MCF10A-Myc^ON^, MCF10A-Myc^OFF^	–	–	Inhibition of IGFBP-6, IGF-1R IGF-I, and IGF-II expression	Not available	–	IGFs/IGF-1R	Downregulation of IGFBP-6 or IGF-I or IGF-II expression levels via siRNAs in breast epithelial cells or knockdown IGF-1R activity on fibroblasts could play an effective role in changing fibroblast mobilization, suppressing TME remodeling and tumor invasion via the IGFs/IGF-1R axis.	De Vincenzo et al., 2019
Triple-negative breast cancer (TNBC)	IGF-1R	+	MDA-MB-231, BT-549	–	–	Inhibition of IGF-1R expression	Not available	–	PI3K-Akt	IGF-1R knockdown via NVP-AEW541, 3-MA, and Atg7 siRNA could induce TNBC cell-protective autophagy and thereby attenuating the efficacy of IGF-1R-modulating therapeutic agents in tumor cells.	Wu W. et al., 2017
Ovarian cancer (OC)	IGF2	+	HEY-T30	–	–	Inhibition of IGF2 expression	Not available	Paclitaxel	IGF	Suppression of the IGF signaling pathway via siRNA could promote sensitivity to paclitaxel in OC cells.	Huang et al., 2010
OC	IGF2	+	HEY-T30	6–8-week-old female athymic nude mice	+	Inhibition of IGF2 expression	Plasmid	Paclitaxel	IGF	IGF2 knockdown via siRNA leading to the suppression of paclitaxel resistance in OC cells.	Brouwer-Visser et al., 2014
Colorectal cancer (CRC)	IGF-1R	+	SW480	–	–	Inhibition of IGF-1R expression	Plasmid	5-Fluorouracil	IGF-1R	Inhibiting CRC cell proliferation and promoting chemosensitization to 5-FU.	Yavari et al., 2010
CRC	IGF-1R	+	SW480	–	–	Inhibition of IGF-1R expression	Not available	–	IGF-1R	Utilizing radioconjugate of IGF-1R siRNA, p-SCN-Bn-DTPA, and 177Lu as radiopharmaceutical to suppress CRC cell proliferation caused by upregulation of IGF-1R via triggering apoptosis.	Fathi et al., 2013
CRC	IGF-1R, IR-A	+	SW480	–	–	Inhibition of IGF-1R and IR-A expression	Plasmid	–	IGF-1R	Inhibiting IR-A expression causing a concomitant promotion of IGF-1R activation through IGF-I and IGF-II, decreasing the formation of IGF-1R: IR-A hybrid receptors, and enhancing IGF-1R homodimer formation in CRC cells.	Brierley et al., 2010
CRC	IGF-1R, PIAS3	+	HT29, HT29-OxR, DLD-1-OxR	–	–	Inhibition of IGF-1R, PIAS3 expression	Not available	Ganitumab, NVP-AEW541, Dasatinib, FOLFOX, CAPOX, FOLFIRI, Oxaliplatin	IGF-1R, AKT, Wnt	Upregulation of PIAS3 could contribute to promoting the expression level of IGF-1R that in turn leading to Wnt pathway activation and thus causing resistance to chemotherapeutic agents. IGF-1R and PIAS3 knockdown via siRNAs leading to the chemotherapy sensitivity in CRC cells.	Codony-Servat et al., 2017
CRC	IGF-1R	+	HCT116	–	–	Inhibition of IGF-1R expression	Plasmid	5-fluorouracil, Cisplatin	IGF-1R, MEK/ERK, PI3K/AKT	IGF-1R knockdown via siRNA could lead to upregulation of miR-497 and activation of PI3K/AKT signaling pathway, which in turn promoting the sensitivity of CRC cells to the chemotherapeutic drugs 5-fluorouracil and cisplatin.	Guo et al., 2013
Gastric carcinoma (GC)	AKT, ERK1, ERK2	+	MGC803, SGC-7901	–	–	Inhibition of AKT, ERK1, and ERK2 expression	Plasmid	–	AKT/ERK	Upregulation of IGF-I could trigger EMT in gastric cancer cells which is accompanied by enhancing ZEB2 expression level. Thus, AKT, ERK1, and ERK2 knockdown via siRNA could reverse IGF-I-induced ZEB2 up-regulation and EMT via promoting the expression of miR-200c.	Li et al., 2014
Pancreatic cancer (PC)	FAK	+	Panc-1, MiaPaca-2	–	–	Inhibition of FAK expression	Adenoviral	–	IGF-1R, FAK	Dual knockdown of FAK and IGF-1R via TAE 226 and siRNA could lead to remarkable suppression of cell viability, reducing ERK and AKT phosphorylation levels, and promoting apoptosis in PC cells which in turn resulting in caspase-3 activation as well as ADP-ribose and PARP cleavage in tumor cells.	Liu et al., 2008
PC	PTEN	+	BxPC-3, SW1990, AsPC-1, MIA PaCa-2, PANC-1	–	–	Inhibition of PTEN expression	Not available	–	IGF-1, PI3K/AKT, NFκB	IGF-1 could trigger tumor cell growth and invasiveness of PC cells leading to promoting activation of PI3K/AKT/NFκB signaling as well as downregulating phosphorylation of PTEN. PTEN knockdown via siRNA could increase PI3K/AKT/NFκB pathway activation and increasing tumor cell proliferation and invasion.	Ma et al., 2010
Prostate adenocarcinoma (PaC)	IGFBP-2	+	DU145, PC3	–	–	Inhibition of IGFBP-2 expression	Not available	Docetaxel	–	Downregulation of IGFBP-2 via siRNA modulating promotion of PTEN activity as well as sensitivity to docetaxel in CaP cells.	Uzoh et al., 2011
Acute myeloid leukemia (AML)	IGF-1R, IR, PI3K	+	U937	–	–	Inhibition of the class Ia PI3K isoforms p110β and p110δ	Plasmid	–	PI3K/AKT, ERK	Targeting isoforms p110β and p110δ via RNAi could reduce AKT activation through IGF-I or insulin, indicating that both PI3K isoforms contributing to the upregulation of IGF-1R or IR in AML cells and improving the sensitivity of tumor cells to chemotherapeutical drugs.	Doepfner et al., 2007
Ewing’s sarcoma (ES)	IGF-1R, EWS/FLI-1	+	TC-71	–	–	Inhibition of IGF-1R and EWS/FLI-1 expression	Not available	R1507	IGF, AKT	Transferring EWS/FLI-1 siRNA leading to upregulation of IGF-BP3 levels and downregulation of IGF-1 and IGF-2 levels and following that reducing p-Akt levels, thereby suppressing signaling via p-IGF-1R. As a result, triggering apoptosis and proliferation inhibition in ES cells.	Huang et al., 2011
Lung Cancer (LC)	IGF-1R	+	A549	–	–	Inhibition of IGF-1R expression	Lomustine-loaded chitosan nanoparticles (ChiNPs)	Doxorubicin	IGF-1R, STAT3	The IGF-1R siRNA/DOX co-delivery system loaded chitosan nanoparticles play an effective role in reducing mmp9, STAT3, and VEGF in tumor cells.	Shali et al., 2018
LC	IGF-1R	+	H460	5–6-week-old female athymic nude mice	+	Inhibition of IGF-1R expression	Not available	Cisplatin	IGF-1R	IGF-1R knockdown via siRNA could upregulate the expression level of IGFBP-3 in tumor drug resistance cells and lead to promoting the sensitivity of LC cells to cisplatin and radiation.	Sun et al., 2012
Non-small cell lung carcinoma (NSCLCs)	IGFBP7	+	PC-9, PC-14	–	–	Inhibition of IGFBP7 expression	Not available	Cisplatin	–	IGFBP7 knockdown via siRNA could play an effective role in promoting resistance to cisplatin as well as upregulating the expression level of MKP-3 in NSCLCs.	Okamura et al., 2012
NSCLCs	IRS-1	+	H1299	–	–	Inhibition of IRS-1 expression	Not available	Cisplatin	DSBs repair and checkpoint	IRS-1 knockdown via siRNA indicating that IRS-1 and ATM expression levels are downregulated by IGF-1 that could contribute to promoting cisplatin resistance in NSCLC cells and blocking the activation of DSBs repair and checkpoint pathways as well as cisplatin-induced γH2AX formation.	Jeon et al., 2008
Renal cell carcinoma (RCC)	IGF-2, HOTTIP	+	A-498, 786-O	–	–	Inhibition of IGF-2, HOTTIP expression	Plasmid	–	IGF-2	LncRNA HOTTIP could contribute as a miR-615 sponge which negatively modulates its target IGF-2. There is a positive association between the expression of HOTTIP and IGF-2 in tumor cells. HOTTIP knockdown via siRNA could remarkably suppress cell growth and carcinogenesis, and promote apoptosis in RCC cells.	Wang Q. et al., 2018
Esophageal squamous cell carcinoma (ESCC)	IGFBP5	+	SLMT-1	–	–	Inhibition of IGFBP5 expression	Not available	Cisplatin	IGF	IGFBP5 knockdown via siRNA indicating Cisplatin resistance in ESCC cells, and thereby upregulation of IGFBP5 could play an important role in promoting sensitivity of tumor cells to chemotherapeutic agents.	Chan et al., 2018
Hepatocellular carcinoma (HCC)	IGF-1R	+	Huh7, Hep3B	6-week-old BALB/c nude mice	+	Inhibition of IGF-1R expression	Lentiviral	–	IGF-1R	IGF-1R knockdown via lentivirus-mediated RNAi could remarkably suppress tumor cell growth and apoptosis through attenuating the expression level of midkine in HCC cells.	Bie et al., 2016

## Discussion

IGFs and the related signal transduction networks partake in the pathogenesis of cancers, diabetes complications, atherosclerosis, PCOS, and other disorders. Meanwhile, these signaling pathways are regulated by hundreds of miRNAs and lncRNAs. Several members of IGF signaling including IGF-I, IGF-II, IGF-1R, and IGFBP-3 are targets of regulation by miRNAs and lncRNAs. Therefore, understanding the complex interplay between these factors is a necessary step in the design of appropriate therapeutic options for these conditions. The importance of this task has been further underscored by the availability of several IGF-modifying modalities including receptor-specific antibodies, inhibitors of receptor kinases, and activators of AMP-activated protein kinases (Pollak, 2008). In addition to these types of therapeutics, a number of alternative medicines act by affecting the expression of IGF-related non-coding RNAs. For instance, bufothionine induces gastric cancer cell apoptosis via up-regulating miR-133a-3p which sponges IGF1R and regulates PI3K/Akt associated production of reactive oxygen species (Hu Z. H. et al., 2020).

The data presented above indicate that most of the IGF-associated lncRNAs exert their roles via modulation of miRNAs. Examples of lncRNA/miRNA interactions in the IGF-related pathways are circ_0014130/miR-142-5p, Linc00319/miR-147a, TUG1/miR-148b, H19/miR-18b, HCP5/miR-27a-3p and DBH-AS1/miR-138. The association between lncRNAs/miRNAs and the IGF system has importance in regenerative medicine as well. IGF1R signaling has been shown to partake in the preservation of stem cell features and improvement of efficiency of stem cell therapy, as IGF1R-expressing stem cells exhibit strong pluripotent or multipotent features (Teng et al., 2018). Therefore, the lncRNA/miRNA-mediated regulation of IGF1R signaling might offer putative modalities for maintaining stem cell features and enhancing the effects of these therapeutics in clinical settings.

IGF-related miRNAs and lncRNAs can be used as potential markers for forecasting the prognosis of cancer. Moreover, expression levels of these transcripts can be used as diagnostic markers for neoplastic conditions. The importance of IGF signaling in the modulation of response of melanoma, ovarian cancer, breast cancer, pancreatic cancer, prostate cancer, colorectal cancer, and several other cancers to chemotherapeutic agents has been validated. Some lncRNAs and miRNAs such as H19, LUCAT1, miR-143, miR-497, and miR-223 are involved in this process. However, the role of other transcripts should be assessed in the upcoming researches. Based on the role of IGF-related miRNAs and lncRNAs in the modulation of response o chemotherapeutic agents, these transcripts are putative targets for the improvement of the response of cancer cells to these agents.

Besides, promoter methylation of IGF-1R, IGF-1, IGF-II, and especially IGFBP-3 in various regions could be associated with cancer prognosis (Supplementary Table 3). Methylation patterns of these promoters are important for the regulation of their expression and could have pivotal clinical implications in various cancers. Re-expression of IGFBP-3 will be really helpful in curing the majority of aggressive tumors and can solve the problem of intratumoral heterogeneity.

Furthermore, employing CRISPR-Cas9 or siRNAs gene editing tools with the aim of knockdown of ectopic expression of target genes including IGF1R, IGF1, IGF2, IGFBP3, and IGFBP-6 can play an important role in attenuating the tumorigenesis characteristics as well as improving response to treatment in various human cancer cells. Utilizing this effective method will pave the way for future clinical advancement.

## Conclusion

The advent of novel genome editing modalities and clarification of the role of epigenetic factors including both genomic marks and non-coding RNAs have raised the possibility of management of human cancers particularly neoplastic disorders with novel therapeutics. Meanwhile, concomitant assessment of expression profile and genomic marks of IGF-related genes using high throughput methods would facilitate appropriate stratification of patients with regards to possible response to each therapeutic option. Further investigations are needed to appraise the clinical application of novel therapeutic modalities that target IGF signaling and related lncRNAs.

### Availability of Data and Materials

The analyzed data sets generated during the study are available from the corresponding author on reasonable request.

## Author Contributions

MT and SG-F supervised the study, wrote the draft, and edited the submission. HS, AA, and MM performed the data collection, designed the tables and figures. All of the authors are contributed equally and fully aware of submission.

## Conflict of Interest

The authors declare that the research was conducted in the absence of any commercial or financial relationships that could be construed as a potential conflict of interest.

## Supplementary Material

Supplementary Table 1 IGF-associated miRNAs in cancers (NNTs: nearby normal tissues).

Supplementary Table 2 Role of different drug inhibitors in suppressing the IGF-1R activity and attenuating tumorigenesis as well as drug resistance in various human cancer cells and promoting response to treatment.

Supplementary Table 3 Epigenetic regulation of different genes associated with the IGF signaling pathway in human cancers.

## Abbreviations

IGFs: insulin-like growth factors
MAPK: mitogen-activated protein kinase
IGFBP: insulin-like growth factor binding proteins
lncRNAs: long non-coding RNAs
miRNAs: microRNAs
IGFBP-3: IGF binding protein-3
EMT: epithelial-mesenchymal transition
NNTs: nearby normal tissues
GBM: Glioblastoma
OC: ovarian cancer
OS: osteosarcoma
HCC: hepatocellular carcinoma
BCa: breast cancer
NPC: nasopharyngeal carcinoma
GC: gastric cancer
NSCLC: non-small cell lung carcinoma
LGG: low-grade gliomas
WT: wilms tumor
RB: retinoblastoma
OSCC: oral squamous cell carcinoma
CRC: colorectal cancer
RCC: renal cell carcinoma
ULM: uterine leiomyoma
PTC: papillary thyroid carcinoma
EC: endometrial carcinoma
M: melanoma
SCCHN: squamous cell carcinoma of head & neck
HUVECs: human umbilical vascular endothelial cells
CHD: coronary heart disease
DR: diabetic retinopathy
PE: preeclampsia
PCOS: polycystic ovary syndrome
PNI: peripheral nerve injury
RA: rheumatoid arthritis
IPF: idiopathic pulmonary fibrosis
NAFLD: non-alcoholic fatty liver disease
SCI: spinal cord injury
AMI: acute myocardial infarction
LDD: lumbar disc degeneration
DMR: differentially methylated region
PaC: pancreatic cancer
TSCC: tongue squamous cell carcinoma
VSMC: vascular smooth muscle cell
TNBC: triple-negative breast cancer
PaC: prostate adenocarcinoma
ACC: adrenocortical carcinoma
GIST: gastrointestinal stromal tumor
HDACi: histone deacetylase inhibitors
NICTH: non-islet cell tumor hypoglycemia
HB: hepatoblastoma
LA: lung adenocarcinoma
MM: multiple myeloma
EOC: epithelial ovarian cancer
DLBCL: diffuse large b-cell lymphoma
EWS: ewing sarcoma
liver CSCs: liver cancer stem cells.
