# Supplementary material for: The Interplay Between Non-coding RNAs and Insulin-Like Growth Factor Signaling in the Pathogenesis of Neoplasia
Source: Front Cell Dev Biol. 2021 Mar 9;9:634512. doi: 10.3389/fcell.2021.634512 (PMC7985092; doi:10.3389/fcell.2021.634512)
Supplement: Supplementary Table 1 — IGF-associated miRNAs in cancers (NNTs: nearby normal tissues). [file Table_1.docx]

Supplementary Table 1 | IGF-associated miRNAs in cancers (NNTs: nearby normal tissues).

| **Type of Cancer** | **miRNA** | **P-Value** | **Model** | **Human Samples** | **Cell Lines** | **Target** | **Pathway** | **Function** | **Ref** |
| --- | --- | --- | --- | --- | --- | --- | --- | --- | --- |
| Glioblastoma (GBM) | miR-15b | P<0.001 | Male BALB/c nude mice | 40 pairs of GBM and adjacent normal tissues | U87, U373, U251, A172, NHAs | IGF-1R | - | Overexpression of miR-15b by targeting IGF-1R could inhibit cell proliferation in vivo, and suppress the proliferation, invasion, cell cycle arrest of U87 and U251 cells in vitro. | Wang J. et al., 2017 |
| Ovarian Cancer (OC) | miR-19a-3p | P<0.05 | Female BALB/nude mice | - | ES-2, SKOV3,  293T | IGFBP-3, Caspase-3 | NF-kB | Overexpression of miR‐19a‐3p by suppressing IGFBP‐3 could enhance the growth and migration of OC cells. | Bai et al., 2019 |
| Osteosarcoma (OS) | miR-26a | P<0.01 | - | 32 pairs of OS and adjacent normal tissues | MG-63, U2OS | IGF-1 | - | miR-26a by targeting IGF-1 could suppress cell proliferation in OS cells and inhibit tumor growth in mouse xenografts. | Tan et al., 2015 |
| Hepatocellular Carcinoma (HCC) | miR-28-5p | P<0.05 | - | 46 pairs of HCC and adjacent normal tissues | HepG2,  HuH-7 | IGF-1 | PI3K/AKT | Downregulation of miR-28-5p by targeting IGF-1 via regulating the PI3K/AKT pathway could reduce tumor proliferation, migration, and early apoptosis in HCC. | Shi and Teng, 2015 |
| Hepatocellular Carcinoma (HCC) | miR-29a-3p | P<0.001 | BALB/c-nude | 63 pairs of HCC and adjacent normal tissues | HepG2, Hep3B, HuH-7, L02,  SMMC-7721 | IGF-1R, CCL5 | - | Overexpression of miR-29a-3p by downregulating IGF-1R could inhibit the growth and migration of HCC cells. | Wang X. et al., 2017 |
| Breast Cancer (BCa) | miR-29,  miR-30 | P<0.05 | C57BL/6 J mice | - | 4 T1 | IGF-1 | AKT | miR-29/30 via targeting IGF-1 could be involved in calorie restriction in both the normal liver and liver with breast cancer metastases. | Shastri et al., 2020 |
| Nasopharyngeal Carcinoma (NPC) | miR-30a | P<0.05 | - | - | CNE2, Hone1 | IGF-1,  E-cadherin, Vimentin | Src | IGF-I could be involved in inducing EMT in CNE2 and HONE-1 cells via Src/miR-30a/E-cadherin axis. | Wang et al., 2016 |
| Breast Cancer (BCa) | miR-99a | P<0.01 | BALB/c nude mice | - | MDA-MB-231 | IGF-1R | - | Overexpression of miR-99a by targeting IGF-1R inhibits proliferation, migration, and invasion of MDA-MB-231 cells. | Xia et al., 2016 |
| Gastric Cancer (GC) | miR-99b-5p | P<0.01 | - | 433 GC tissue samples and 50 normal tissues | SGC-7901, MKN-45,  GES-1 | IGF-1R, Cyclin-D1, CDK4/6 | - | Overexpression of miR-99b-5p/203a-3p by targeting IGF-1R could reduce GC cell proliferation and cell cycle progression in vitro in GC. | Wang Z. et al., 2018 |
| Osteosarcoma (OS) | miR-100 | P<0.01 | - | 20 pairs of OS and adjacent normal tissues | HOS, U-2OS, Saos-2, MG-63,  NHOst | IGF-1R, HIF-1α, Caspase-3 | PI3K/AKT, MAPK/ERK | Overexpression of miR-100 via decreasing IGF-1R through the PI3K/AKT and MAPK/ERK pathways could inhibit cell proliferation, migration, and invasion function of U-2OS, and MG-63 cells. | Liu Y. et al., 2016 |
| Breast Cancer (BCa) | miR-100 | P<0.001 | Balb/C mice | 32 pairs of BCa and adjacent normal tissues | 67NR, 4T1, MDAMB231, T47D, MCF7, SKBR3 | IGF-2 | mTOR | Overexpression of miR-100 by suppressing IGF-2 and regulating the mTOR signaling pathway could inhibit tumor growth. | Gebeshuber and Martinez, 2013 |
| Glioma | miR-128 | P<0.05 | - | - | Hs683,  M059K, U87MG | IGF-1, MMP, ROS, LC3-I/II, Caspase-3 | mTOR | Downregulation of miR-128 by targeting IGF-1 and activating mTOR signaling could result in temozolomide-induced glioma cell apoptotic death. | Chen et al., 2016 |
| Gastric Cancer (GC) | miR-133a-3p | P<0.01 | BALB/c nu/nu mice | - | MGC-803, BGC-823, 293T | IGF-1R, ROS, Sal, NAC | PI3K/AKT | Overexpression of miR-133a-3p by increasing the IGF-1R could inactivate the PI3K/AKT pathway in GC. | Hu Z. H. et al., 2020 |
| Non-Small Cell Lung Carcinoma (NSCLC) | miR-135a | P<0.05 | - | 138 pairs of NSCLC and adjacent normal tissues | A549, H460, H1299 | IGF-1, VEGF, bFGF,  IL-8 | PI3K/AKT | Overexpression of miR-135a by targeting the IGF-1 via activating the PI3K/AKT signaling could inhibit cell proliferation, migration, invasion, and tumor angiogenesis in NSCLC. | Zhou Y. et al., 2017 |
| Low-Grade Gliomas (LGG) | miR-138 | P<0.05 | Male BABL/c nude mice | 89 pairs of LGG and adjacent normal tissues | Res186,  Res259 | IGF2BP2 | - | Overexpression of miR-138 by targeting IGF2BP2 could suppress tumor development in vivo, and inhibit proliferation and invasion of LGG cells in vitro. | Yang Y. et al., 2020 |
| Glioma | miR-139 | P<0.001 | Male BALB/Nude mice | 24 glioma and 10 normal tissues | HCN-2, U251, U87MG | IGF-1R, AMY-1, PGC-1β | AKT | Overexpression of miR-139 via AKT signaling by targeting IGF-1R could suppress glioma cell migration and invasion in vitro, and inhibit glioma growth in vivo. | Wang H. et al., 2017 |
| Wilms Tumor (WT) | miR-140-5p | P<0.05 | - | 23 pairs of WT and adjacent normal tissues | G401,  WT-CLS1,  293T | IGF-1R, TGFBR-1 | SMAD2/3, AKT | Overexpression of miR-140-5p via regulating the TGFBR-1/SMAD2/3 and IGF-1R/AKT signaling pathways could inhibit tumor proliferation and progression in WT. | Liu Z. et al., 2019 |
| Retinoblastoma (RB) | miR-145 | P<0.01 | - | - | WERI-RB1, SO-RB50,  Y79 | IGF-1R | - | Overexpression of miR-145 by targeting IGF-1R could suppress the proliferation and induce the apoptosis of Y79 cells. | Chen Z. et al., 2018 |
| Oral Squamous Cell Carcinoma (OSCC) | miR-148a | P <0.01 | - | 110 pairs of OSCC and adjacent normal tissues | SCC-15,  HOK | IGF-1R | ERK/MAPK, MEK1, JNK | Overexpression of miR-148a via inhibiting the ERK/MAPK pathway by targeting IGF-1R could inhibit the OSCC cell proliferation, migration, and invasion in vitro. | Jia et al., 2020 |
| Breast Cancer (BCa) | miR-152 | P<0.05 | - | 18 pairs of BCa and adjacent normal tissues | MCF7, T47D,  MDA-MB-231, MCF10A,  MDA-MB-453, | IGF-1,  β-catenin, PKM2 | - | Overexpression of miR-152 by targeting and inhibiting both β-catenin and PKM2 expressions via IGF-1 could suppress cell proliferation in BCa cells. | Wen et al., 2017 |
| Hepatocellular Carcinoma (HCC) | miR-155 | P<0.0001 | - | HCC (n=23), biopsies from healthy livers (n=10) | HuH-7, HepG2 | IGF-2, IGF-1R, IGFBP-3 | - | Overexpression of miR-155 by upregulating IGF-2 and IGF-1R and by downregulating IGFBP-3 could increase the proliferation, migration, and clonogenicity of HCC. | El Tayebi et al., 2015 |
| Non-Small Cell Lung Carcinoma (NSCLC) | miR-155-5p | P<0.05 | Female nude mice | - | A549,  H1975 | IGFBP1, | FOXO3a, STAT3 | Interaction between miR155-5p and FOXO3a via targeting IGFBP-1 could lead to the inactivation of STAT3, which then leads to suppressing human lung cancer cell growth. | Zheng et al., 2018 |
| Wilms Tumor (WT) | miR-155-5p | P<0.001 | - | 87 pairs of WT and adjacent normal tissues | G401,  SKNEP-1,  HK-2 | IGF-2 | PI3K/AKT/mTOR | Overexpression of miR-155-5p by inactivating the PI3K/AKT/mTOR pathway via binding and inhibiting IGF-2 could inhibit cell proliferation, migration, and invasion in G401 and SK-NEP-1 cells. | Luo X. et al., 2020 |
| Colorectal Cancer (CRC) | miR-184 | P<0.05 | - | 32 pairs of CRC and adjacent normal tissues | HCT116, HT29, SW620, FHC, 293T | IGF11R | - | Overexpression of miR-184 by negatively regulating IGF-1R could suppress proliferation, migration, and invasion in CRC cells. | Wu G. et al., 2017 |
| Colorectal Cancer (CRC) | miR-185 | P<0.05 | - | - | RR−HCT116 | IGF-1R, IGF-2 | - | Overexpression of miR-185 by targeting the IGF-1R/IGF-2 axis could increase the sensitivity of CRC cells to ionizing radiation leading to induced apoptosis. | Afshar et al., 2018 |
| Glioma | miR-188 | P<0.05 | - | 19 pairs of glioma and adjacent normal tissues | U87, U251, U118, LN229, LN18, NHA, 293T | IGF2BP2 | - | Overexpression of miR-188 by targeting IGF2BP2 could inhibit the cell proliferation, migration, and invasion of glioma. | Ding et al., 2017 |
| Renal Cell Carcinoma (RCC) | miR-193b | P<0.05 | SPF BALB/C nude mice | 45 pairs of RCC and adjacent normal tissues | HK2, Caki-1, 786-O, ACHN | IGF-1R | - | Overexpression of miR-193b by directly targeting IGF-1R could inhibit the proliferation, migration, and invasion in RCC. | Chen J. et al., 2019 |
| Colorectal Cancer (CRC) | miR-195 | P<0.01 | - | 90 pairs of CRC and NNTs | SW480, SW620 | IGF2BP2, RAF1 | - | Overexpression of IGF2BP2 via blocking miR-195-mediated degradation in SW480 cells could regulate RAF1 mRNA stability. | Ye S. et al., 2016 |
| Colorectal Cancer (CRC) | miR-197 | P<0.05 | - | 21 pairs of CRC and NNTs | HCT8, HCT116, SW480 | IGFBP-3 | - | Overexpression of miR-197 by targeting IGFBP-3 could increase migration and invasion in CRC cells. | Zhou et al., 2018 |
| Uterine Leiomyoma (ULM) | miR-197 | P<0.01 | - | 30 pairs of ULM tissues and normal myometrium | - | IGFBP-5 | - | Overexpression of miR-197 via targeting IGFBP5 could inhibit cell proliferation in human ULM cells. | Ling et al., 2015 |
| Breast Cancer (BCa) | miR-199a-5p | P<0.001 | - | - | MCF-7,  MDA-MB-231 | IGF-1, DDR1 | PI3K/AKT | IGF-1 by decreasing miR-199a-5p and upregulating DDR1 via the PI3K/AKT pathway could be involved in suppressing proliferation and migration of BCa cells. | Mata et al., 2016 |
| Gastric Cancer (GC) | 203a-3p | P<0.01 | - | 433 GC tissue samples and 50 normal tissues | SGC-7901, MKN-45,  GES-1 | IGF-1R, Cyclin-D1, CDK4/6 | - | Overexpression of miR-99b-5p/203a-3p by targeting IGF-1R could reduce GC cell proliferation and cell cycle progression in vitro in GC. | Wang Z. et al., 2018 |
| Papillary Thyroid Carcinoma  (PTC) | miR-204-5p | P<0.05 | Male BALB/c nude mice | 16 pairs of PTC  and adjacent normal tissues | TCP-1, BCPAP, 293T | IGFBP-5 | - | Overexpressing of miR-204-5p via regulating IGFBP5 could enhance the induction of cell cycle arrest and apoptosis and inhibit PTC cell tumorigenicity in vivo. | Liu et al., 2015b |
| Renal Cell Carcinoma (RCC) | miR-214 | P<0.001 | - | - | HK2, HRPTEC, ACHN, 786-O, A498, RCC4 | IGF-1, GSK3β | mTORC1, AKT | Overexpression of miR-214 by decreasing IGF-1R protein levels could inhibit AKT kinase activity in both types of RCC. | Das et al., 2016 |
| Hepatocellular Carcinoma (HCC) | miR-216b | P<0.0001 | Male BALB/c nude mice | 150 pairs of HCC and adjacent normal tissues | L02, CT26,  SMMC-7721, HepG2, 97-H, LM3, PLC/PRF/5, | IGF2BP2, HBx, Caspase-3/8, Bax, Bcl-2 | AKT, mTOR, ERK | Overexpression of miR-216b by downregulating IGF2BP2 could inhibit cell proliferation, migration, and invasion in HCC. | Liu et al., 2015a |
| Breast Cancer (BCa) | miR-320a | P<0.01 | - | 12 pairs of BCa and adjacent normal tissues | MDA-MB-231 | IGF-1R, Cyclin-D1, caspase-3/9, Bax | AKT | Overexpression of miR-320a by the PI3K/AKT pathway via targeting IGF-1R could inhibit tumor cell growth and invasion of human BCa cells. | Guan et al., 2018 |
| Osteosarcoma (OS) | miR-223 | P<0.001 | - | 50 pairs of OS and adjacent normal tissues | HOS, MG-63 | IGF-1R, Caspase-3/9, Bax, Bcl-2 | - | Overexpression of miR-223 by targeting IGF-1R could inhibit OS proliferation and induce apoptosis. | Xiao et al., 2019 |
| Hepatocellular Carcinoma (HCC) | miR-342-3p | P<0.01 | Male nude mice | - | HepG2, MHCC97H, 293T | IGF-1R, GLUT1 | PI3K/AKT/GLUT1 | Overexpression of miR-342-3p by suppressing IG F-1R could inhibit glycolysis expression and cell proliferation in hepatoma cells. | Liu et al., 2018d |
| Oral Squamous Cell Carcinoma (OSCC) | miR-375 | P<0.001 | - | 44 pairs of OSCC and adjacent normal tissues | SCC-4 | IGF-1R | - | Overexpression of miR-375 by targeting the IGF-1R could induce cell cycle arrest and enhance radiosensitivity in OSCC cells. | Zhang et al., 2017 |
| Non-Small Cell Lung Carcinoma (NSCLC) | miR-379 | P<0.05 | - | 49 pairs of NSCLC and adjacent normal tissues | H460, A549, H1299,  SPC-A-1, BEAS-2B | IGF-1R | AKT, ERK | Overexpression of miR-379 by targeting IGF-1R could suppress cell proliferation, migration, and invasion in NSCLC. | Zhou F. et al., 2017 |
| Endometrial Carcinoma  (EC) | miR-381 | P<0.05 | - | 45 pairs of EC and adjacent normal tissues | HEC-1B, KLE,  HEC-59, AN3CA, Ishikawa | IGF-1R | AKT, ERK | Overexpression of miR-381 by targeting IGF-1R could suppress the proliferation and invasion of EC cells. | Tu et al., 2018 |
| Melanoma  (M) | miR-425 | P<0.01 |  | 15 pairs of M and adjacent normal tissues | A375,  SK-MEL-28 | IGF-1, Twist1,  AP-1 | PI3K-AKT | Overexpression of miR-425 via suppressing the PI3K-AKT pathway activated by IGF-1 could suppress cell proliferation and metastasis of melanoma. | Liu et al., 2015c |
| Colorectal Cancer (CRC) | miR-448 | P<0.01 | - | 28 pairs of CRC and adjacent normal tissues | HCT116, HT29, SW480, LOVO, SW620 | IGF-1R | - | Overexpression of miR-448 via regulating IGF-1R could inhibit proliferation, colony formation, migration, and invasion in CRC cells. | Li B. et al., 2016 |
| Hepatocellular Carcinoma (HCC) | miR-455-5p | P<0.001 | - | 82 pairs of HCC and adjacent normal tissues | HepG2, Hep3B, Bel-7404, HuH-7 | IGF-1R, GLUT1 | AKT/GLUT1 | Overexpression of miR-455-5p via IGF-1R/AKT/GLUT1 pathway by targeting IGF-1R could inhibit HCC cell growth, migration, and invasion. | Hu et al., 2019 |
| Non-Small Cell Lung Carcinoma (NSCLC) | miR-485-5p | P=0.0217 | Male BALB/c nude mice | 87 pairs of NSCLC and adjacent normal tissues | A549,  NCI-H460, NCI-H1299, BEAS-2B | IGF2BP-2, TGF-β | - | Overexpression of miR-485-5p via targeting IGF2BP2 could restrain the growth and metastasis of NSCLC in vivo and in vitro. | Huang et al., 2018 |
| Hepatocellular Carcinoma (HCC) | miR-486-5p | P<0.01 | - | HCC (n=20), healthy controls (n=10) | HuH-7 | IGF-1R, STAT3,  c-Myc | mTOR | Overexpression of miR-486-5p by downregulating IGF-1R via the mTOR/STAT3 axis could increase the HCC suppression. | Youness et al., 2016 |
| Colorectal Cancer (CRC) | miR-491-5p | P<0.001 | Male BABL/c nude mice | 80 pairs of CRC and adjacent normal tissues | HCT116, HCT8, HT29, SW620, SW480, FHC | IGF-2 | - | Overexpression of miR-491-5p by targeting IGF2 could suppress proliferation both in vitro and in vivo CRC. | Lu et al., 2019 |
| Oral Squamous Cell Carcinoma (OSCC) | miR-495 | P<0.05 |  | 13 pairs of OSCC and adjacent normal tissues | TCA-83,  SCC-4, SCC-9, HN-5, HOK | IGF-1,  ZO-1,  E-cadherin, a-SMA, Vimentin | AKT | Downregulation of miR-495 by targeting IGF-1 could suppress proliferation, migration, and invasion, and EMT–related proteins of OSCC cells. | Wang et al., 2019d |
| Hepatocellular Carcinoma (HCC) | miR-505 | P<0.001 | - | 60 pairs of HCC and adjacent normal tissues | HepG2, HuH-7, SMMC-7721, Bel-7404 | IGF-1R, GLUT1 | AKT/GLUT1 | Overexpression of miR-505 by targeting IGF-1R could suppress the proliferation and colony formation in HCC cells. | Ren et al., 2019 |
| Gastric Cancer (GC) | miR-598 | P<0.05 | Balb/c nude mice | 90 pairs of GC and adjacent normal tissues | MKN-28,  BGC-823, AGS, GES-1, 293T | IGF-1R | ERK1/2, AKT | Overexpression of miR-598 by targeting IFG-1R and via suppressing the ERK1/2 and AKT phosphorylation level could suppress cell proliferation, migration, invasion, and induce cell cycle arrest in GC cells. | Liu et al., 2018c |
| Non-Small Cell Lung Carcinoma (NSCLC) | miR-615-3p | P<0.01 | Nude mice | NSCLC (n=24), paratumor  tissues (n=15) | H1975, A549, HCC827, PLC/PRF/5 | IGF-2 | - | Overexpression of miR-615-3p by targeting IGF-2 could suppress NSCLC growth and lung metastasis . | Liu J. et al., 2019 |
| Hepatocellular Carcinoma (HCC) | miR-615-5p | P<0.01 | - | Peripheral blood sample from HCC patients (n=130) and healthy controls (n=35) | NKs | IGF-1R, TNF-α, NKG2D, ULBP2 | - | Overexpression of miR-615-5p via inhibiting IGF-1R could reduce NKs cytotoxicity and the cytotoxic markers such as TNF-α, and perforins. | Rahmoon et al., 2017 |
| Breast Cancer (BCa) | miR-1193 | P<0.00031 | - | 39 pairs of BCa and adjacent normal tissues | MDA-MB-231,  MDA-MB-468, MDA-MB-435, SKBR3, MCF-7 | IGF2BP2 | - | Overexpression of miR-1193 by targeting IGF2BP2 could inhibit proliferation and invasion of human BCa cells. | Li et al., 2017 |
| Colorectal Cancer  (CRC) | miR-1260b | P<0.01 | - | 30 pairs of CRC and NNTs | HCT116, SW480 | IGF-1, PDCD4 | AKT,  ERK | Overexpression of miR-1260b by targeting the PDCD4/IGF-1 axis could reduce the apoptotic rate and induce CRC cell proliferation in HCT116 cells. | Yao et al., 2018 |
| Squamous Cell Carcinoma of Head & Neck (SCCHN) | miR-1275 | P<0.01 | - | 15 pairs of SCCHN and NNTs | PCI-4, PCI-37 | IGF-1R | - | Overexpression of miR-1275 by upregulating IGF-1R and CCR7 could promote cell migration, invasion, and proliferation in SCCHN. | Liu et al., 2018b |
| HCC | miR-1275 | P<0.001 | - | HCC (n=22), healthy controls (n=19) | HuH-7 | IGF2BP1, IGF2BP2, IGF2BP3 | - | Overexpression of miR-1275 via decreasing in IGF2BP1, IGF2BP2, and IGF2BP3 mRNA expression could inhibit viability, proliferation, wound-healing, and clonogenicity of HuH-7 cell. | Fawzy et al., 2015 |
| Breast Cancer (BCa) | miR-3941 | P<0.001 | - | 37 pairs of BCa and adjacent normal tissues | MDA-MB-231,  MCF-10 | IGF-1,  E-cadherin,  N-cadherin, Vimentin | - | Overexpression of miR-3941 via targeting IGF-1 could decrease BCa cells viability and suppress cell migration and invasion. | Dang et al., 2017 |
| Colorectal Cancer  (CRC) | miR-6165 | P<0.0001 | - | 14 pairs of CRC and NNTs | SW480, HCT116, NT2 | IGF-1R | PI3K/AKT, RAS/  MAPK | Overexpression of miR-6165 could reduce IGF-1R expression and enhance apoptosis in SW480 cells. | Hassanlou et al., 2020 |
| Non-Small Cell Lung Carcinoma (NSCLC) | miR-12528 | P<0.01 | Balb/c nude mice | 20 pairs of NSCLC and adjacent normal tissues | WI-38, WI-38 VA13, A549,  BEAS-2B, NCIH1299,  SK-LU-1,  NCI-H596, NCI-H226,  SK-MES-1,  NCI-H460 | IGF-1R | AKT | Overexpression of miR-12528 by targeting IGF-1R could inhibit proliferation, apoptosis, migratory activity, tumorigenesis, and metastasis of lung cancer in vivo. | Jeon et al., 2018 |

Supplementary Table 2 | Role of different drug inhibitors in suppressing the IGF-1R activity and attenuating tumorigenesis as well as drug resistance in various human cancer cells and promoting response to treatment.

| **Treatment** | **Combined Treatment** | **Type of Cancer or Disease** | **In vitro** | **Cell line** | **Animal/Human** | **In vivo** | **Target** | **Effect** | **Pathway** | **Ref** |
| --- | --- | --- | --- | --- | --- | --- | --- | --- | --- | --- |
| AG1024 | Gefitinib, Metformin, siRNA | Non-Small Cell Lung Carcinoma | + | H1975 | 6-week-old female BALB/cA-nu mice | + | IGF-1R | Inhibitor | IGF-1R, AKT/BIM | Pan et al., 2018 |
|  | Trastuzumab | Breast cancer (BCa) | + | MDA-MB-231, H2N, HR2 | - | - | IGF-1R | Inhibitor | - | Cornelissen et al., 2008 |
|  | AG1478, PP2, siRNA | BCa | + | MCF-7 | - | - | IGF-1R, ERα/IGF-1R, cSrc, EGF-R | Inhibitor | IGF-R, EGF-R | Song et al., 2010 |
|  | Crizotinib | Cervical cancer | + | HeLa | - | - | IGF-1R, ALK | Inhibitor | IGF-1R, ALK | Sin et al., 2019 |
| AZD3463 | Zoledronic acid | Breast cancer | + | MDA-MB-231BO | 4-week-old nude mice | + | IGF-1R | Inhibitor | IGF-1R, PI3K-AKT | Hu G. F. et al., 2020 |
|  | SN-38, Vorinostat, Temozolomide | Ewing sarcoma | + | A4573, TC32 | - | - | ALK, IGF-1R | Inhibitor | IGF-1R , STAT3/AKT/MAPK | Sampson et al., 2015 |
| BMS-536924 | Temozolomide | Glioma | + | M059K, U87MG, M059K-R, U87MG-R | Nud/nud mice | + | IGF-1R/IR | Inhibitor | IGF-1R/IR | Zhou, 2015 |
|  | 3-aminobenzamide | Ovarian cancer | + | OVCAR-3, OVCAR-4, SKOV-3, TOV-81D | - | - | IGF-1R | Inhibitor | IGF-1R, PARP | Beauchamp et al., 2009 |
|  | Alloxan, Metformin | Breast cancer | + | 4T1 | 6-week-old female BALB/c mice | + | IGF-1R/IR | Inhibitor | - | Dool et al., 2011 |
|  | - | Esophageal cancer, Adenocarcinoma | + | TE8, OE33, OE19 | - | - | IGF-1R/InsR, AKT, MAPK | Inhibitor | IGF-1R | Adachi et al., 2014 |
|  | - | Leukemia | + | ML-1, K562, U937 | - | - | IGF-1R/IR | Inhibitor | IGF, AKT, MEK1/2 | Hendrickson et al., 2009 |
| BMS-754807 | Letrozole, Tamoxifen, 4-Hydroxytamoxifen, Fulvestrant | Breast cancer | + | MCF-7/AC-1 | 4-6-week-old female ovariectomized BALB/c athymic nude mice | + | IGF-1R/InsR | Inhibitor | IGF, ERK/AKT | Hou et al., 2011 |
|  | SiRNA | Colorectal cancer | + | HT55, SK-CO-1, SNU175, HCC-56, NCI-H508, LS411N, SW1417, COLO-678, GP5D, RKO PM | - | - | IGF-1R/IR | Inhibitor | IGF-1R/IR, PI3K/AKT, MEK/MAPK | Huang et al., 2015 |
|  | Gemcitabine | Pancreatic ductal adenocarcinoma | + | AsPC-1, BxPC-3,MIA PaCa-2, Panc-1 | 4–6-week-old athymic female nude mice | + | IGF-1R/IR, AKT | Inhibitor | IGF | Awasthi et al., 2012 |
|  | Platinum-based chemotherapeutics, Cisplatin | Non-Small Cell Lung Carcinoma | + | A549, NCI-H358 | - | - | IGF-1R/IR, AKT | Inhibitor | IGF-1R/IR, PI3K/AKT | Franks et al., 2016 |
|  | - | Pediatric high-grade glioma | + | GaMG, DK-MG, U87-MG, LN-18, pHGG KNS42, SF188 | - | - | IGF-1R | Inhibitor | - | Simpson et al., 2020 |
|  |  |  |  |  |  |  | γH2AX, 53BP1, RAD51 foci | Interrupt DDR |  |  |
| Ceritinib (LDK378) | Alectinib, Ceritinib, TAE684, ASP3026, AP26113 | Lymphoma | + | Karpas299WT, Karpas299CR, Karpas299CR^G1269A^ | - | - | ALK/MET | Inhibitor | IGF-1R | Li Y. et al., 2019 |
|  | Dasatinib | Osteosarcoma | + | 6 primary tumor cells isolated from OS patients | The 16-year-old female patient suffered from OS | + | IGF | Inhibitor | IGF | Beck et al., 2020 |
|  | Vinblastine, Vinca alkaloids, Doxorubicin, Actinomycin D | HGNET-BCOR brain tumor | + | PhKh1 | - | - | IGF1R, AKT | Inhibitor | IGF1R/AKT | Vewinger et al., 2019a |
|  | Arsenic trioxide (ATO), Linsitinib | Brain tumor | + | 225ZL | Blood and brain tissue samples | + | IGF-1R, INSR | Inhibitor | IGF, SHH, WNT, Notch | Russo et al., 2019 |
| Ginsenoside Rg5 | - | Alzheimer's disease | - | - | Wistar rats | + | BDNF, IGF-1, ChAT activity | Activator | - | Chu et al., 2014 |
|  |  |  |  |  |  |  | Inflammatory cytokines TNF-α and IL-1β, AChE activity, Aβ deposition, COX-2, iNOS | Inhibitor |  |  |
|  | - | Angiogenesis and vasorelaxation | + | HUVECs | 7-week-old male mice (C57BL/6J, eNOS^-/-^, and ApoE^-/-^), Sprague-Dawley rats | + | IGF-1R | Activator | IGF-1R, NO/cGMP, PI3K/AKT/eNOS, MEK/ERK, Src/FAK/paxillin | Cho et al., 2015 |
| GSK1838705A | - | Glioma | + | U87MG | 7-8-week-old female athymic nude mice | + | IGF-1R | Inhibitor | IGF | Zhou et al., 2015 |
|  | Regorafenib, Linsitinib (OSI-906) | Hepatocellular carcinoma | + | PLC/PRF/5, HepG2, HLF | - | - | IGF-1R | Inhibitor | PI3K/AKT, MAPK | Refolo et al., 2017 |
|  | - | Lymphoma | + | NIH-3T3/LISN, NIH-3T3-hIR | 8-12-week-old female nu/nu CD-1 mice | + | IGF-1R, ALK | Inhibitor | IGF-1R | Sabbatini et al., 2009 |
|  | BMS-754807, U0126, siRNA | Colorectal cancer | + | HT29, HCT116, SW480, LoVo, RKO | 4-5-week-old male and female athymic nude mice | + | IGF-1R, AKT2 | Inhibitor | IGF-1R | Wang Q. et al., 2020 |
|  |  |  |  |  |  |  | p70S6K1, MEK1/2 | Activator |  |  |
| GSK1904529A | - | Glioma | + | U87MG | 6-8-week-old female Balb/cA-nu mice | + | IGF-1R | Inhibitor | IGF/IGF-1R | Vewinger et al., 2019 |
|  | - | Osteosarcoma | + | Saos-2, MG-63, OB-6, Primary OS | 8-9-week-old female nude mice | + | IGF-1R | Inhibitor | IGF-1R | Fei et al., 2017 |
| Linsitinib (OSI-906) | Sirolimus, Everolimus | Adrenocortical cancer | + | H295R, HAC15 ACC | - | - | IGF-1R/IR | Inhibitor | IGF, mTOR | De Martino et al., 2019 |
|  | - | Nasopharyngeal Carcinoma | + | CNE-1, CNE-2, SUNE-1, 5-8F, 6-10B | - | - | IGF-1R | Inhibitor | IGF-I/IGF-1R, PI3K/AKT, MAPK/ERK | Wang et al., 2019f |
|  | NVP-ADW-742 | Ewing sarcoma | + | A4573, TC32, TC71 | Male NOD (SCID)-IL-2Rg^null^ mice | + | IGF-1R/IR-α | Inhibitor | IGF/PI3K/AKT/mTOR | Lamhamedi-Cherradi et al., 2016 |
|  | Tamoxifen | ER-positive breast cancer | + | MCF-7, T47D | - | - | IGF-1R/InsR | Inhibitor | IGF-1R/InsR, PI3K/MAPK | Kruger et al., 2020 |
|  | - | Gastrointestinal stromal tumors | - | - | 20 GIST patients | + | IGF-1R | Inhibitor | - | Von Mehren et al., 2020 |
| NVP-ADW742 | Imatinib (STI571) | Small cell lung cancer | + | H526, H146, H209, WBA, MRC-5 | - | - | IGF-1R | Inhibitor | IGF-1R, PI3K/AKT | Warshamana-Greene et al., 2004 |
|  | Temozolomide | Medulloblastoma | + | Daoy | - | - | IGF-1R, PI3K, P38, GSK-3β, AKT | Inhibitor | IGF-1R | Zhou et al., 2011 |
|  | Cytarabine (Ara-C) | Acute myeloid leukemia | + | HL-60 | - | - | IGF-1R | Inhibitor | IGF-1R | He et al., 2010 |
|  | Etoposide, Carboplatin | Small cell lung cancer | + | H526, H146, WBA, H209 | - | - | IGF-1R | Inhibitor | PI3K/AKT | Warshamana-Greene et al., 2005 |
| NVP-AEW541 (AEW541) | 3-methoxyamphetamine (3-MA), siRNA | Triple-negative breast cancer | + | MDA-MB-231, BT-549 | - | - | IGF-1R | Inhibitor | PI3K/AKT | Wu W. et al., 2017 |
|  | - | Orthotopic pancreatic cancer | + | HPAF-II, BxPC-3 | 8-week-old male athymic nude mice (BALB/cnu/nu) | + | IGF-1R, IRS-1, ERK, AKT, IGFBP-3, STAT3 | Inhibitor | IGF-1R, STAT3, AKT, ERK | Moser et al., 2008 |
|  | ICR62, LY294002, U0126 | Colorectal cancer | + | Colo2, Colo13, CCL247/HCT-116, CCL218/HT-29, CCL221/DLD-1, CCL225/HCT-15, CCL227/SW620, CCL228/SW480, CCL231/SW48, CCL235/SW837, CCL244/HCT-8/HRT-18 | - | - | IGF-1R | Inhibitor | PI3K/AKT, MEK/MAPK | Cunningham et al., 2008 |
|  | Dasatinib | Glioblastoma multiforme | + | U87, T98G, U373, LN229, A172, LNZ308 | - | - | IGF-1R, ERK, AKT | Inhibitor | IGF-1R | Premkumar et al., 2010 |
|  |  |  |  |  |  |  | AIF release, Bax oligomerization | Activator |  |  |
| NVP-TAE 226 (TAE226) | - | Esophageal cancer | + | SEG-1, KOB-13 | - | - | IGF-1R, FAK, mTOR, AKT, p70S6K, S6 | Inhibitor | IGF-1R, FAK/PI3K/mTOR, PI3K/AKT/mTOR | Wang et al., 2008 |
|  | PF-562,271 | Ewing sarcoma | + | TC71, SK‐ES‐1, RD‐ES, RD, 293T, SU‐CCS‐1, HS‐Os‐1 | 8-14‐week-old female NOD/SCID/JAK3 null mice | + | IGF-1R, FAK | Inhibitor | IGF-1R, FAK, EGFR, BDNF, TGF‐β | Moritake et al., 2019 |
|  | - | Breast cancer | + | MDA-MB-231, ST2, RAW264.7 | 5-week-old female BALB/c nude mice | + | IGF-1R, FAK, RANKL | Inhibitor | IGF-1R, FAK | Kurio et al., 2011 |
|  | - | Glioma | + | U87, U87/EGFR, U87/vIII, U251, U251/EGFR, U373, LN229, LN382T, LN18, LN308, A172, D54, SNB19, U343 | 6-8-week-old male nude mice | + | IGF-1R, FAK, AKT, MAPK | Inhibitor | IGF-1R, FAK | Liu et al., 2007 |
|  | - | Barrett's esophageal adenocarcinoma | + | SEG-1, FLO-1, BIC-1 | 8-week-old female BALB/cAJc1-nu/nu mice | + | IGF-1R, FAK, AKT | Inhibitor | AKT-BAD-caspase | Watanabe et al., 2008 |
|  | - | Non-Small Cell Lung Carcinoma | + | HCC827, HCC4006, NCI-H3255, NCI-H1975, NCI-H820, NCI-H1819, NCI-H1666, NCI-H1395, NCI-H2228, NCI-H1648, NCI-H1993, NCI-H838, NCI-H1299 | 4–6-week-old female BALB/c-nu/nu nude mice | + | IGF-1R, FAK, EGFR | Inhibitor | EGFR | Otani et al., 2015 |
| Picropodophyllin (AXL1717) | ASP3026 | NPM-ALK+ T-cell lymphoma | + | Karpas 299, DEL, SR-786 | 6–8-week-old female C.B-17 SCID mice | + | IGF-1R, ALK, STAT3 | Inhibitor | - | George et al., 2019 |
|  | Imatinib Mesylate, Cisplatin, 5-fluorouracil, Doxorubicin | Uveal melanoma | + | OCM-1, OCM-3, OCM-8, 92-1 | 10-week-old pathogen-free SCID mice | + | IGF-1R, VEGF | Inhibitor | - | Economou et al., 2008 |
|  | Sorafenib | Hepatocellular carcinoma | + | HLF, PLC/PRF/5, HUVEC | - | - | IGF-1R | Inhibitor | - | Tomizawa et al., 2014 |
|  | JNJ-10198409 | Glioblastoma multiforme | + | U87-MG (U87), A172, T98G (T98), LN-229 | - | - | IGF-1R, PDGFR, AKT, ERK1/2 | Inhibitor | IGF-1R, PDGFR, AKT, ERK1/2 | Carrasco-Garcia et al., 2018 |
|  | siRNA | Medulloblastoma | - | - | Medulloblastoma-prone mice (Ptc1^F1-2m/WT^, Trp53^F2-10/F2-10^ , Pax7^ICNm/WT^) | + | IGF-1R | Inhibitor | IGF | Ohshima-Hosoyama et al., 2010 |
|  |  |  |  |  |  |  | Caspase-3 | Activator |  |  |
| PQ401 | - | Osteosarcoma | + | U2OS, 143B | 5 tissues of OS patients | + | IGF-1R | Inhibitor | IGF-1R | Qi et al., 2019 |
|  | - | Glioma | + | U87MG | 6-week-old male nude mice (nu/NOD/SCID  strains) | + | IGF-1R | Inhibitor | IGF-1R, PI3K/AKT | Zhou et al., 2016 |
|  | - | Skin cancer | - | - | 7-week-old Swiss albino mice | + | IGF-1R, MMP9, syndecan-1, fascin-1 | Inhibitor | IGF-1R | Alyoussef, 2020 |
|  | - | Breast cancer | + | MCF-7, MCNeuA | FVB/N-TgN(MMTVneu)202 female mouse strain | + | IGF-1R | Inhibitor | IGF-1R, AKT | Gable et al., 2006 |
|  | Gefitinib | Head and neck squamous carcinoma | + | SCC-25, Cal27 | - | - | IGF1R, EGFR | Inhibitor | IGF1R, PI3K/AKT, MEK/ERK | Jameson et al., 2011 |

Supplementary Table 3 | Epigenetic regulation of different genes associated with the IGF signaling pathway in human cancers

| **Type of Cancer or Disease** | **Target** | **In vitro** | **Cell line** | **Animal/Human** | **In vivo** | **Epigenetic alteration** | **Treatment** | **Pathway** | **Function** | **Ref** |
| --- | --- | --- | --- | --- | --- | --- | --- | --- | --- | --- |
| Breast cancer (BCa) | IGFBP-3 | + | MCF-7, T47D, Hs578T, MDA-MB-231 | - | - | Promoter CpG island hypermethylation | 5-Aza-2′-deoxycytidine (AZA), siRNA | - | In ER-α positive cells AZA epigenetically could promote the expression level of p53 and induce demethylation of the IGFBP-3 promoter, whilst in ER-negative cells, AZA upregulates the expression level of AP2-α as well as IGFBP-3. | Zeng et al., 2013 |
| Colorectal cancer (CRC) | IGFBP-3 | - | - | Nurses’ Health Study (N = 121,700 women followed since 1976), Health Professionals Follow-Up Study (N = 51,500 men followed since 1986) | + | Promoter CpG island hypermethylation | - | P53 | IGFBP3 promoter methylation could negatively accompany by microsatellite instability in CpG island methylator phenotype-high tumors in colorectal p53-negative cancer cells. | Kawasaki et al., 2007 |
| Esophageal carcinoma (EC) | IGF-1R, IGFBP-3, IGF-1 | - | - | 264 patients (case group) whom EC radical resection was performed, and 283 healthy individuals (control group) | + | Promoter CpG island hypermethylation | - | IGF | Promoter methylation of IGF-1, IGF1R, and IGFBP3 was significantly accompanied by EC incidence as well as Clinicopathological Characteristics of patients like histologic differentiation degree and TNM staging. | Ye P. et al., 2016 |
| Gastric cancer (GC) | IGFBP-3 | + | AGS, KATO-III, MKN-1, MKN-28, MKN-45, MKN-74, N87, SNU-1, SNU-5, SNU-16, SNU-216, SNU-484, SNU-601, SNU-620, SNU-638, SNU-668, SNU-719, YCC-1, YCC2, YCC-3, YCC-7, OCUM-2M | 482 tissue samples of GC patients | + | Promoter CpG island hypermethylation | - | - | IGFBP-3 promoter methylation measuring by MS-PCR plays an effective role in detecting the survival rate in particular subgroups of patients suffering from early-stage gastric cancer undergoing curative procedure. | Kim et al., 2015 |
| GC | IGFBP-3 | - | - | 94 gastric adenocarcinoma samples, and 43 normal gastric mucosa specimens | + | Promoter CpG island hypermethylation | - | - | The expression level of IGFBP-3 was considerably higher n diffuse-type tumors situated in the non-cardia region compared with the cardia. | Gigek et al., 2010 |
| Non-islet cell tumor hypoglycemia (NICTH) | IGF-II | - | - | A 53-year-old man with metastatic hemangiopericytoma | + | Methylation of the IGF2/H19 imprinting control regions  (In one allele within exon 9 of the IGF2 gene a 19-nucleotide deletion was detected, for the H19 gene, in exon 5 one allele includes an RsaI restriction enzyme recognition site) | - | - | Loss of IGF2 gene imprinting and various promoter utilization trigger upregulation of IGF-2 leading to LOI of IGF2, but not H19, in the NICTH metastatic tumor. | Lawson et al., 2009 |
| Hepatoblastoma (HB) | IGFBP-3 | + | HUH6, JCRB, HepT1, HepT3, HepG2, HUH7 | 45 liver tumor and 7 adjacent normal tissues | + | Promoter CpG island hypermethylation | 5-Aza-2′-deoxycytidine (AZA) | IGF | IGFBP3 promoter methylation could occur in a high proportion in metastatic HB with vascular invasion. Overexpression of the IGFBP3 gene in HB cells via 5-aza-2’-deoxycytidine could play an effective role in attenuating colony formation, migration, and invasion. | Regel et al., 2012 |
| Hepatocellular carcinoma (HCC) | IGF-II | + | HepG2, Huh-7 | 48 tumor tissue from patients with HBV infection-positive/HCV infection-negative HCC, and 11 patients with both HBV and HCV infection-negative HCC | + | Hypomethylation of CpG island of P3 and P4 promoters | - | - | Hepatitis B virus X protein could play an effective role in MBD2-HBx-CBP/p300 complex formation through interaction with MBD2 and CBP/p300 which could, in turn, lead to the hypomethylation and upregulation activity of the IGF-II-P3 and P4 promoters and that CBP/p300-mediated acetylation of histones H3 and H4 can contribute to triggering activation of these two promoters. | Liu et al., 2015d |
| HCC | IGF-II | - | - | 34 matched adjacent nontumor specimens and 8 normal adult liver specimens | + | Hypomethylation of CpG island of the P4 promoter | - | - | Exotic hypomethylation of IGF-II P4 promoter could play an effective role in upregulation activity of P4 transcription expression within the alteration of a premalignant liver lesion to HCC in a Chinese Population. | Tang et al., 2006 |
| HCC | IGFBP-3 | + | HepG2, Hep3B, PLC/PRF/5, HLE, HuH-7 | 12 HCC tissue samples | + | 13 methylated CpG sites in the promoter region of the IGFBP-3 (Cytosines at nucleotide 178, 189, 196, 200, 208, 223, 227, 234, 243, 249, 255, 261, 274) | 5-Aza-2′-deoxycytidine (AZA) | - | Methylated CpG sites of IGFBP-3 promoter could suppress the expression level of IGFBP-3 in human HCCs. IGFBP-3 plays a remarkable role downstream of many growth suppressors and apoptosis-inducing cascades and thereby triggering tumorigenesis of HCC. | Hanafusa et al., 2002 |
| HCC | IGFBP-3 | + | HepG2 | - | - | Promoter CpG island hypermethylation | - | - | Since 4 out of 7 p53 consensus sequences located upstream of the IGFBP-3 promoter can be vital for the p53 triggered expression of IGFBP-3, hypermethylation of these sequences in promoter could inhibit IGFBP-3 expression induced by p53 in HepG2 cells. | Hanafusa et al., 2005 |
| HCC | IGF-II | - | - | 80 pairs of HCC and adjacent normal tissues  42 Sprague-Dawley rats (4–6 weeks) | + | Unmethylation of CpG island of the P3 promoter | - | - | Upregulation of oncogenic IGF-II expression level and changing methylation status of IGF-II P3 CpG site due to none methylation band in the HCC tissue could result in enhancing malignancy of hepatocytes. | Tai et al., 2019 |
| HCC | IGF-II | + | Hep3B, Huh7, SNU449, PLC5 | 228 HCC tumor samples, 168 paired non-tumor adjacent  cirrhotic liver samples, and 10 normal liver samples  15-day-old C57BL/6 male mice  p19^Arf-/-^ mice (male C57BL/6 background) | + | Hypomethylation of both alleles of the strongest  fetal promoters (P3 and P4) and hypermethylation of  the adult promoter P1 | Sorafenib | IGF | Overexpression of IGF2 via DNA methylation de-regulation of its fetal promoter could promote liver tumor formation with the hepatic expression of MYC and AKT1 through IGF1 receptor signaling activation and thereby enhancing cell proliferation and migratory effects. | Martinez-Quetglas et al., 2016 |
| Lung adenocarcinoma (LA) | IGFBP-4 | - | - | 76 pairs of LA and adjacent normal tissues | + | Promoter CpG island hypermethylation | - | - | IGFBP-4 promoter methylation causes epigenetic silencing and thereby could remarkably downregulate the expression level of IGFBP-4 in LA cells leading to disruption of the mechanism of IGFBP-4-mediated growth suppression. Besides, methylation of EGR-1-binding regions can be related to IGFBP-4 gene silencing. | Sato et al., 2011 |
| Non-Small Cell Lung Carcinoma (NSCLC) | IGFBP-3 | + | H-460, H-23, H23R, H460R, H1299, H727, HT-29, PANC1 | 25 pairs of NSCLC and adjacent normal tissues | + | Promoter CpG island hypermethylation | Cisplatin, siRNA | IGFIR/AKT | Both IGFBP-3 promoter methylation and IGF-1R/AKT phosphorylation can be seen merely in cisplatin-resistant NSCLC patients, suggesting that IGFBP-3 methylation-derived defect may modulate cisplatin resistance in NSCLC patients via upregulation of the IGFIR/AKT pathway. Moreover, a combination of IGFBP-3 promoter methylation with AKT, IGFIR, or EGFR activation status could be applied to detect sensitivity or resistance to cisplatin in NSCLC specimens. | Cortes-Sempere et al., 2013 |
| NSCLC | IGFBP-3 | + | H23, H23R, H460, H460R | 36 pairs of NSCLC and adjacent normal tissues | + | Promoter CpG island hypermethylation | Cisplatin, siRNA, 5-Aza-2′-deoxycytidine (AZA), Trichostatine A (TSA) | - | IGFBP-3 has an opposite effect on the progression rate of cisplatin resistance NSCLC cells. Cisplatin could induce alterations in IGFBP-3 expression modulated by the attainment of promoter hypermethylation which in turn could enhance cisplatin resistance to tumor cells via different biological cascades. | De Caceres et al., 2010 |
| NSCLC | IGFBP-3 | + | H596, A549, H1944, H460, H358, SK-MES-1, H226B, H1299, H661, H441, H322, H226Br, Calu-6 | 63 NSCLC samples and 39 nonmalignant adjacent lung tissues | + | Promoter CpG island hypermethylation (mutation in the Sp-1 site (CCCGCC) in the pGL2-D1708 construct which CCC in the Sp-1-binding site was altered to (AAA)) | 5-Aza-2′-deoxycytidine (AZA) | - | Methylation of the Sp-1/Sp-3-binding element in the IGFBP-3 promoter could play an effective role in the binding of Sp-1, MeCP2, and HDAC. Transcriptional deficiency of IGFBP-3 expression could trigger via interference with Sp-1 transactivation by MeCP2 in NSCLC cells with the methylated promoter. | Chang et al., 2004 |
| Skin cancer (melanoma) | IGFBP-3 | + | WM35, WM1341, WM902B, WM9, WM45.1, MeWo, LOX, FEMX-1, WM983 | 6 benign nevi, 17 primary malignant melanomas, and 40 metastases tissue samples | + | Promoter CpG island hypermethylation | 5-Aza-2′-deoxycytidine (AZA),  siRNA | IGF, PI3K/AKT, MAPK/ERK1/2 | Overexpression of IGFBP-3 during melanoma development may due to the dual function via triggering an effective role in both apoptosis and proliferation. Various procedures including epigenetic silencing via promoter methylation as well as the involvement of MAPK/ERK1/2 and PI3-K/AKT signaling pathways. | Øy et al., 2010 |
| Multiple myeloma (MM) | IGFBP7 | + | U266, KMS-12-BM, OPM-2, NCI-H929, SK-MM-1, RPMI8226 | Specimens of cohorts of MM patients including CD138+ purified MM cells of newly diagnosed, previously untreated myeloma patients of the Heidelberg-Montpellier group (HM; n = 247), and the independent Little-Rock (LR; n = 701) | + | Promoter CpG island hypermethylation | 5-Aza-2′-deoxycytidine (AZA) | - | IGFBP7 can be related to prognostically adverse chromosomal aberrations (t(4;14) and gain of 1q21), MMSET expression, and thereby leading to promoting myeloma cell proliferation. In stromal cells in the vicinity of MM cells the expression of IGFBP7 inhibited which could release the full potential of osteoblast inhibitory molecules such as activin A, resulting in enhancing myeloma bone disease. Thus upregulation of IGFBP7 could lead to a lower possibility of myeloma bone disease. | Bolomsky et al., 2015 |
| Epithelial ovarian cancer (EOC) | IGFBP-3 | + | OVTW59-P0, P4, A549, H1299, 293T | 60 pairs of OEC and adjacent normal tissues | + | Promoter CpG island hypermethylation | 5-Aza-2′-deoxycytidine (AZA), Pifithrin-α (PFT-α) | - | IGFBP-3 promoter hypermethylation could cause IGFBP-3 knockdown in the lack of p53 overexpression can be related to tumor development. Thus, the biological environment of IGFBP-3 promoter methylation is rigorously controlled in a way that un-methylated IGFBP-3 promoter region and wt p53 are some vital factors to sustain a homeostatic condition. | Torng et al., 2009 |
| EOC | IGF-II | - | - | 211 pairs of OEC and adjacent normal tissues | + | Hypermethylation of CpG island of P2 and P3 promoters, and hypomethylation of the P4 promoter | - | - | From three promoters situated in a CpG island, the expression level of IGF-II can be affected via methylation alterations which resulting in inhibiting promoter-specific expression. P3 methylation and expression could play an effective role in ovarian cancer compared with other promoters. | Qian et al., 2011 |
| Prostate cancer (PCa) | IGFBP-3 | + | DU145, PC3, LNCaP PCa | - | - | Promoter CpG island hypermethylation  Hyperacetylation of H3 and no change in the acetylation of H4 | 5-Aza-2′-deoxycytidine (AZA), siRNA, Docetaxel | - | Promoted glucose concentrations could lead to promoting IGFBP2 through enhancing the acetylation of histones related to IGFBP2 gene promoter and thus resulting in suppressing the effect of docetaxel and reducing response to chemotherapy at inducing apoptosis. | Biernacka et al., 2013 |
| PCa | IGF-II | - | - | 141 pairs of PCa and adjacent normal tissues | + | Hypermethylation of CpG island of P2 and P3 | - | IGF | IGF2 expression in PCa is mostly regulated via differential methylation of promoter-specific and not dependent on imprinting status of the IGF2-H19 locus. Various methylation statuses of the site 4A within the P4 IGF2 promoter could have a crucial role in IGF2 expression level in PCa cells. | Küffer et al., 2018 |
